# Context matters (but how and why?) A hypothesis-led literature review of performance based financing in fragile and conflict-affected health systems

**DOI:** 10.1371/journal.pone.0195301

**Published:** 2018-04-03

**Authors:** Maria Paola Bertone, Jean-Benoît Falisse, Giuliano Russo, Sophie Witter

**Affiliations:** 1 ReBUILD & Institute for Global Health and Development, Queen Margaret University, Edinburgh, United Kingdom; 2 Centre of African Studies, University of Edinburgh, Edinburgh, United Kingdom; 3 Centre for Primary Care and Public Health, Queen Mary University, London, United Kingdom; University of Virginia, UNITED STATES

## Abstract

Performance-based financing (PBF) schemes have been expanding rapidly across low and middle income countries in the past decade, with considerable external financing from multilateral, bilateral and global health initiatives. Many of these countries have been fragile and conflict-affected (FCAS), but while the influence of context is acknowledged to be important to the operation of PBF, there has been little examination of how it affects adoption and implementation of PBF. This article lays out initial hypotheses about how FCAS contexts may influence the adoption, adaption, implementation and health system effects of PBF. These are then interrogated through a review of available grey and published literature (140 documents in total, covering 23 PBF schemes). We find that PBF has been more common in FCAS contexts, which were also more commonly early adopters. Very little explanation of the rationale for its adoption, in particular in relation with the contextual features, is given in programme documents. However, there are a number of factors which could explain this, including the greater role of external actors and donors, a greater openness to institutional reform, and lower levels of trust within the public system and between government and donors, all of which favour more contractual approaches. These suggest that rather than emerging despite fragility, conditions of fragility may favour the rapid emergence of PBF. We also document few emerging adaptations of PBF to humanitarian settings and limited evidence of health system effects which may be contextually driven, but these require more in-depth analysis. Another area meriting more study is the political economy of PBF and its diffusion across contexts.

## Introduction

Performance based financing (PBF) schemes typically aim to improve health services by providing bonuses to service providers (usually facilities, but often with a portion paid to individual staff) based on the verified quantity of outputs produced, modified by quality indicators. In many cases there is a division of functions between regulation, purchasing, fund-holding, and service delivery [1–3]. PBF schemes have been expanding rapidly across low and middle income countries in the past decade, focussed on maternal and child health services, with considerable external financing from multilateral, bilateral and global health initiatives [3]. The initial rationale was to align provider incentives with public policy objectives, especially linked to the Millennium Development Goals—boosting uptake of services such as supervised deliveries, antenatal care and immunisation [4]. More recently, focus has shifted to the need for greater focus on quality of care, as increased utilisation was not reflected in equivalent health gains [5] and also on how PBF might be catalytic of wider health system changes [1,6,7]. There is a growing literature on PBF’s impact on different health care indicators [8–14] and also on its ‘theory of change’ (understanding the mechanisms through which PBF may or may not work) [15,16], as well as analysing the extent to which PBF is distinct from previous approaches [17].

It is clear from the early literature that PBF is unlikely to be a homogenous intervention and that its modalities and effects will be highly dependent on context [13]. However, the literature on PBF has been surprisingly thin in its discussion of how different contexts may influence the adoption, adaption and design, implementation and effects of PBF programmes [18]. Additionally, PBF has been promoted as the preferred health financing (and health system) intervention of many donors, raising concerns which echo earlier debates about the promotion of standardised models by development partners [19]. Monitoring and evaluation strategies by leading funding agencies rarely aim to isolate or examine contextual factors [20,21], despite evaluation reports which highlight that PBF will work differently in different contexts [22]. However, specific contextual features, and in particular those related to fragile, conflict-affected states (FCAS), are likely to have particular impact on the adoption, adaption, implementation and health system effects of PBF.

The term ‘FCAS’ is an increasingly used definition [23,24] covering a very diverse group of states disrupted by violence, armed conflict, natural disasters, and all sorts of governance crises [25]. There is a growing body of literature [26] lending support to the idea that the analysis of health systems in such circumstances needs to pay close attention to their political, economic as well as social contexts, as these determine critical distortions.

This article examines specifically the influence of context on PBF programmes in relation to FCAS. There are a number of reasons for this choice of focus. The first is that many of the early PBF schemes emerged in FCAS countries (such as initially in Cambodia, and then Rwanda, Burundi and Democratic Republic of the Congo) and it is interesting to understand why that might have been. Secondly, the burden of ill-health is increasingly focused in FCAS and so the role of PBF in addressing these health needs is particularly relevant to unpack. Finally, to the extent to which there has been discussion about PBF in FCAS settings, the arguments have been conflicting—with some arguing that PBF is unlikely to be effective in environments with, for example, low ability to absorb risk and weak information systems [27,28], while others point out that precisely in situations of weak institutions there is more potential for PBF to re-align relationships and improve accountability [29]. Furthermore, a recent agenda setting exercise on priority themes for research on health care in FCAS listed understanding the opportunities and challenges for PBF in FCAS as one of the priority research questions [30].

This article’s objective is to interrogate existing grey and published literature on how the FCAS context influences the adoption, adaption, implementation and health system effects of PBF in order to support or refute a set of hypotheses about their interaction. By ‘context’, we understand, in this paper, the differences that exist between countries in terms of socio-economic structure and political organisation, including the way the health system is organised. As our focus is on FCAS, we are particularly interested in fault lines within society, economy, and political system that are inherited from the period of conflict.

## Methods

### Development of hypotheses

The authors started by developing hypotheses about how FCAS contexts might interact with PBF, to be tested against the current published empirical evidence. These hypotheses mainly draw on the framework developed by Witter et al. in 2013 [1] and elements hinted at in the limited literature on PBF and FCAS [29,31,32]. We do not necessarily suggest that they are exhaustive, rather they proved to be useful to frame the results. Other insights also arose from our analysis and further work may propose and test other hypotheses.

Concerning adoption, the hypotheses we looked at included the ideas that FCAS might:

1 Allow more entry for external players (e.g. donors and NGOs) to influence policy innovations such as PBF—so increasing the prevalence and possibly fragmentation (if multiple players) of PBF programmes in FCAS;
2 Let those external players drive (largely or partly) the reform process, at least in the initial stage;
3 With top-level managers thinner on the ground and more dependent on external funders, they are likely to be especially open to international influences / ‘new’ models;
4 FCAS societies may also have lower levels of interpersonal trust that make formal, contractual, arrangements less likely to be resisted / more likely to appear a solution for everybody;
5 Have higher fears of misappropriation / misallocation of funding, making PBF an appealing solution because of its apparent control mechanisms;
6 Have higher local/regional interests to satisfy and weaker central institutions, making the ‘hyper-devolution’ (health facilities having greater autonomy in management) an appealing option;
7 Reduce organised resistance (through weaker labour organisations, for example) to such innovations—leading to increased prevalence but also possibly swifter scale-up.

Interestingly, all of these hypotheses seem to work in the direction of increasing the likelihood of PBF being introduced in FCAS.

In terms of design and adaption, we reviewed the hypotheses that:

8 With reduced system capacity to manage and more players, possibly not well coordinated, there would be increased challenges of uniform scale up in FCAS—with more variety and local adaptation.

Finally, in relation to implementation and health system effects, a number of hypotheses emerge—it is interesting to note that here expectations about the potential success of PBF in FCAS are more mixed. Hypotheses include that:

9 With reduced capacity to fund, FCAS would generally face higher sustainability challenges for PBF;
10 Looser hierarchical controls and less reliable funding flows to lower levels both make PBF an attractive modality as a way for the centre to regain leverage; they do however rely on strong purchasing skills at different levels and without these PBF will be less predictable in its health system effects.

**Fig 1:**
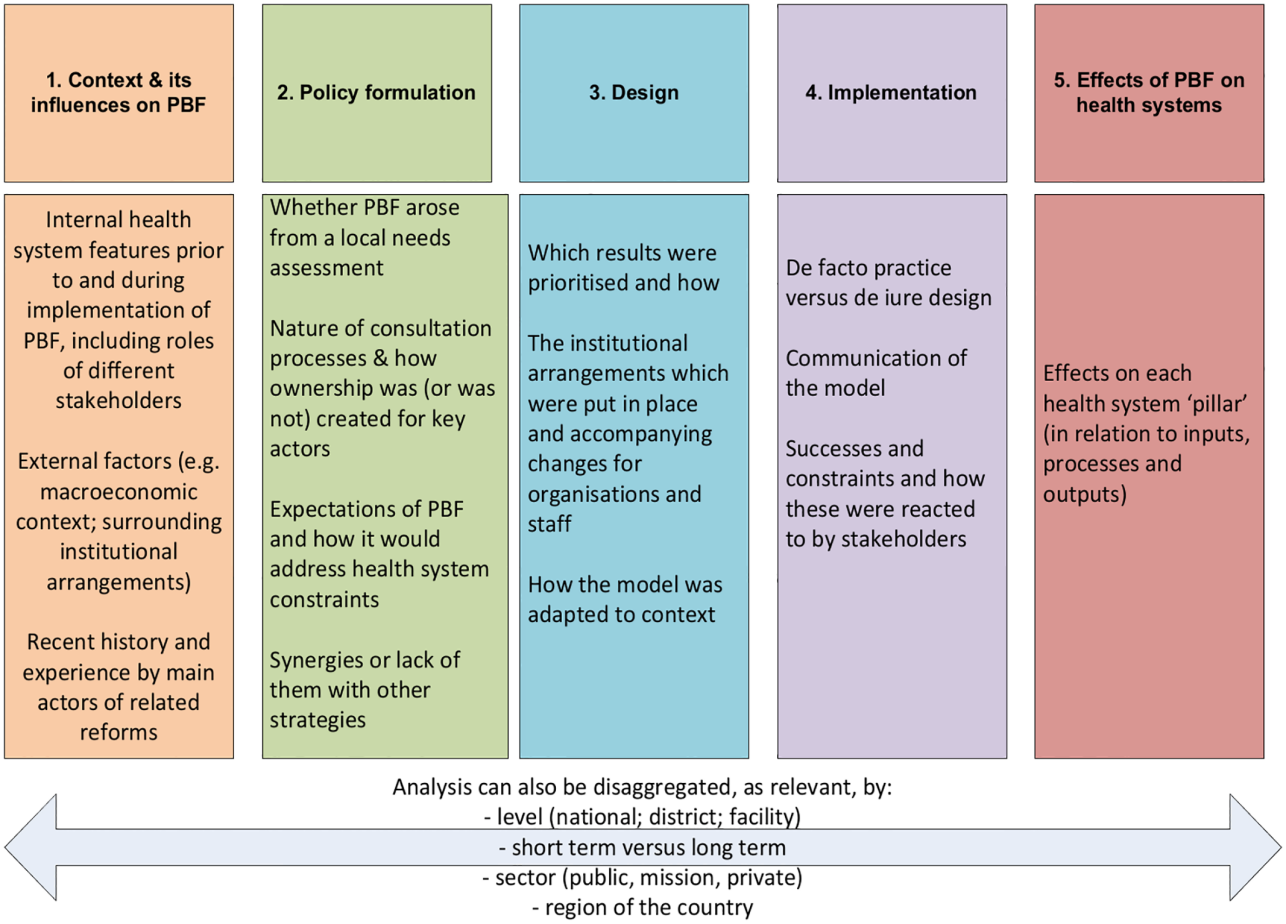
The five domains for understanding PBF-health systems interactions—A framework. Source: [1].

The second analytical phase of our work consisted in gathering evidence from a literature review, in order to examine these expectations and to populate an existing framework developed to assess the interactions between PBF and health systems [1]. The framework starts with a focus on the context in which PBF is adopted and then looks at policy adaption and design, followed by implementation and effects on the six health system pillars (Fig 1).

### Inclusion and exclusion criteria

Inclusion and exclusion criteria for the literature review were defined. We included PBF programmes in the health sector (exclusively), which are defined as supply-side payments targeting managers and providers, usually including volume and quality of healthcare indicators [1]. In order to maintain some homogeneity of intervention, we did not include ‘contracting’ approaches that are also part of the ‘family’ of Results-based Financing (RBF) schemes such as ‘performance-based contracting’, ‘management contracting’, or ‘contracting-out’ [2]. We also did not include demand-side programmes such as conditional cash transfers (CCTs) and vouchers.

Countries were included in the review based on their classification as FCAS in the World Bank’s harmonized list of fragile situations [23] for any year over the last decade, as this was the period in which most PBF programmes were developed in low and middle income countries. The combined list for 2007–2017 includes 53 countries (S1 Table).

We were aware that much of the evidence on PBF is ‘grey’ and so used a very broad definition of literature, to include published academic articles, but also published and unpublished documents, reports, presentations, blog posts etc., with the proviso that they focused on PBF in the FCAS countries, with particular attention to any documents which included a description of the context and of features related to fragility. The review included documents in English, French and Portuguese.

### Search strategy

First, an online literature search was run which included:

- published literature, searched on Google Scholar using key words such as “results-based financing” combined with health and fragility;
- the websites of DfID, WHO and World Bank, which were searched using key words which included “results/performance based financing” combined with context terms (e.g. country names), fragility and health care;
- RBF-focussed websites were searched manually by 27 March 2017. These included:
  - The World Bank’s RBF website (https://www.rbfhealth.org/) (all the 110 webpages of links to documents/resources that were available);
  - DFID’s website (https://www.gov.uk/government/publications?departments%5B%5D=department-for-international-development) using the keywords “performance- based financing” or “results- based financing” + “context” (first 20 pages of results);
  - WHO’s website (http://www.who.int/publications/en/), using the keywords “performance- based financing” or “results- based financing” + “context”;
  - The discussions in the Google Group of the PBF Community of Practice (CoP) (https://groups.google.com/forum/#!forum/performance-based-financing), using the key word ‘context’, in both French and English. The PBF CoP has more than 2,000 members, including PBF experts and practitioners, implementers, policy-makers, donors and researchers with a broad geographical representation and a focus on sub-Saharan Africa.
  - All the resources and blog-posts of the website Health Financing in Africa (http://www.healthfinancingafrica.org/resources.html);
  - The national RBF/PBF portals for each country: Cameroon (http://www.fbrcameroun.org/), CAR (http://www.pbfcar.org/), Burundi (http://www.fbpsanteburundi.bi/), Nigeria (http://pbfnigeria.org), Chad (http://www.fbrtchad.org/, DR Congo (http://www.fbrsanterdc.cd/), and Sierra Leone (http://pbfsierraleone.org/);
  - The websites of Cordaid, a major PBF implementer/funder (https://www.cordaid.org) and SINA Health, a consultancy firm that worked with Cordaid on many PBF projects (http://www.sina-health.com/).

Secondly, a consultation with experts involved in PBF was launched via the PBF CoP using the website *Collectivity* (http://www.thecollectivity.org/en/projects/26). The objectives of our research were laid out and experts asked to share documents describing the PBF programmes in their countries, and in particular, the reasons why the PBF scheme was introduced, its objectives and the challenges it aims/aimed to address, and the context in which it was introduced. Implementation or procedural manuals, project documents, interim reports on implementation, working papers, and internal analysis were solicited.

### Data gathered

In total, 140 documents were reviewed, covering 23 countries. Table 1 gives detail of the documents reviewed by country, type and source. On average, we reviewed 6 documents per country, ranging from 14 for the DR Congo and 13 for Burundi to 1 for Djibouti. In terms of type of documents, the majority are academic papers, either published or unpublished (28, 20%), PBF operational manuals (25, 18%) and research reports (22, 16%). PBF programme documents (18, 13%) and evaluations (16, 11%) are also relevant among the documents retrieved. The remaining are operational reports (7, 5%) or other miscellaneous documents (24, 17%). In terms of sources, the majority of the documents (59, 42%) were retrieved via the CoP consultation and the experts who shared a range of documents and information with us, while the remaining documents were retrieved through the web (53, 38%) and literature searches (28, 20%).

**Table 1:** Documents reviewed by country, type and source.

Country	Num. of documents reviewed	Type of document	Sources
Afghanistan	7	Academic papers (4) PBF programme document (2) Operational reports (1)	Literature search (4) Web search (2) CoP/expert consultation (1)
Burundi	16	Academic papers (6) Working papers (3) Research reports (4) Operational report (2) PBF operational manual (1)	Web search (11) Literature search (3) CoP/expert consultation (2)
Cambodia	8	Academic papers (3) PBF programme documents (2) Research reports (3)	Literature search (3) Web search (5)
Cameroon	7	PBF operational manual (1) PBF programme documents (2) Research reports (2) Academic papers (2)	Web search (4) Literature search (2) CoP/expert consultation (1)
Central African Republic (CAR)	8	PBF operational manual (1) PBF programme evaluation (1) Research reports (2) Programme documents (4)	Web search (4) Literature search (1) CoP/expert consultation (3)
Chad	5	PBF operational manual (1) PBF programme evaluations (2) Academic papers (2)	Web search (1) Literature search (2) CoP/expert consultation (2)
Comoros	6	PBF programme evaluations (4) PBF operational manual (1) PBF strategic document (1)	CoP/expert consultation (6)
Congo	6	PBF operational manual (2) PBF strategic document (1) Powerpoint presentations (3)	Web search (3) CoP/expert consultation (3)
Côte d’Ivoire	5	PBF protocols and operational manuals (3) National PBF strategy (1) Donor project document (1)	Web search (1) CoP/expert consultation (4)
Djibouti	1	PBF operational manual	CoP/expert consultation
DR Congo	14	Academic papers (3) Working papers (3) Research reports (2) Operational report (4) Impact evaluation (1) PBF operational manual (1)	Web search (6) Literature search (3) CoP/expert consultation (5)
The Gambia	5	PBF operational manual Baseline evaluations (3) Donor project document (1)	Web search (3) CoP/expert consultation (2)
Guinea	3	PBF operational manual (1) PBF programme document (1) PBF programme evaluation (1)	CoP/expert consultation
Guinea-Bissau	1	PBF programme document (1)	CoP/expert consultation (1)
Haiti	4	Academic papers (1) PBF programme documents (2) Research reports (1)	Web search (3) Literature search (1)
Laos PDR	7	Academic paper PBF programme document (2) Research report (2) PBF operational manual (2)	Web search (4) Literature search (1) CoP/expert consultation (2)
Liberia	5	Research report (1) PBF programme evaluation (1) PBF operational manual (1) Academic paper (unpublished) (1) Technical brief (1)	Web search (1) Literature search (1) CoP/expert consultation (3)
Mali	8	PBF operational manuals (2) PBF programme evaluation (1) Research report (1) PBF programme document (2) Policy briefs (2)	Literature search (1) CoP/expert consultation (7)
Nigeria	5	PBF operational manual (1) Academic papers (2) Academic paper and presentation (unpublished) (2)	Web search (1) Literature search (2) CoP/expert consultation (2)
Rwanda	11	PBF operational manual (1) Academic papers (6) Research papers (4)	Web search (8) Literature search (2) CoP/expert consultation (1)
Sierra Leone	6	PBF operational manual (1) PBF programme evaluations (2) Academic papers (2) Blog (1)	Web search (1) Literature search (2) CoP/expert consultation (3)
Tajikistan	3	PBF operational manual (2) PBF programme evaluations (1)	Web search (2) CoP/expert consultation (1)
Zimbabwe	6	PBF operational manual (1) PBF programme evaluations (3) Other programme evaluation (1) Academic paper (1)	CoP/expert consultations (6)

### Data analysis

Data were extracted from each document using a series of pre-identified, inductive themes, which are derived from the conceptual framework and adapted to the aim of this study, with a focus on exploring the influence of the context, and in particular of the fragility features of the context, on the adoption and implementation of PBF (Table 2).

**Table 2:** Thematic codes for data extraction and analysis.

Main themes	Sub-themes
***Description of PBF programme***	*Overview of PBF and operational detail (e*.*g*., *dates of pilots/programs*, *funders*, *geographical coverage*, *type of services and facilities covered*, *etc*.*)*
**Context**	Nature of conflict/fragility
	Historical features
	Socio-cultural features
	Political features
	Governance and administrative structures
	Burden of disease
	Health system organisation
**Policy formulation**	Role of external actors (e.g., donors and non-governmental organisations (NGOs))
	Role of internal actors (within health sector and outside—e.g. Ministry of Finance or Presidency)
	Interplay between actors
**Design of PBF**	Specific contextual issues/challenges PBF aims to address
	Adaptation of PBF design to context
**PBF implementation**	Phases of PBF implementation (if relevant)
	Overall operational challenges (in particular in relation to contextual variables)
	Operational challenges in uniform implementation within country
	Changes/adaptations of PBF during implementation
	Political support to PBF during implementation and scale-up (or failure to scale-up)
	Institutional support/challenges during implementation and scale-up
	Financial sustainability of implementation
**Effect on health system, with reference to contextual and FCAS features**	Evidence of impact on health outcomes
	Evidence of impact on governance
	Evidence of impact on healthcare financing
	Evidence on impact on HRH
	Evidence on impact on drugs and equipment
	Evidence on impact on health information system
	Evidence on impact on service delivery

Each researcher was responsible for the review of documents referring to a set of countries. Data extraction was done manually into an Excel spread sheet organised by themes (columns) and countries (rows). This allowed us to summarise and synthetize information, compare and contrast different experiences, interpret data in relation to contexts and map emerging patterns across countries. Preliminary findings were discussed between the research team before drafting the article.

## Results

### Exploring patterns of PBF distribution

We found documents on PBF programmes (on-going or implemented over the period) in 23 out of the 53 FCAS countries identified (43%) [We are aware of a small scale, simple version of PBF implemented in South Sudan (Upper Nile) by Cordaid with World Bank funding, between 2013 and 2016, with some interruptions due to political crisis and violence. However, we were not able to retrieve and review any documents on it, and therefore that project is not included in our analysis]. The World Bank’s *Performance-Based Financing Toolkit* reports that in 2015 there were 34 PBF schemes, at either pilot or national level, among the 51 countries of sub-Saharan Africa [3]. Of these, 19 (56%) are implemented in countries that are included in the FCAS list (note that there are 27 countries included in the FCAS list among the 51 countries of sub-Saharan Africa). Looking at the list of countries with PBF schemes in 2006, all are FCAS (Rwanda is included only on the OECD list). This indicates an earlier and higher rate of adoption of PBF among FCAS countries, although the difference is not substantial overall.

More striking is the pattern in timing of the introduction of PBF between FCAS and non-FCAS settings. Before 2006, the *PBF Toolkit* identifies only three countries in sub-Saharan Africa which had PBF programmes (Rwanda, Burundi and the DR Congo) [3], while our analysis suggests that PBF existed at small scale also in Cameroon and Côte d’Ivoire. Interestingly, all these countries are included in the FCAS list, suggesting that PBF may have been introduced earlier in fragile and post-conflict settings, often as a response to the need of health system’s reorganization in the recovery phase. Additionally, PBF programmes seem to have been scaled-up at national level earlier in FCAS settings compared to other low and middle income countries. Indeed, the first (and for the time being, only) three countries to have introduced PBF nation-wide are Rwanda (2008), Burundi (2010) and Sierra Leone (2011), all FCAS countries. We also note that the implementation of the early PBF programmes follows the introduction of the ‘contracting’ approach in other FCAS settings, such as Cambodia (also the setting of an early PBF experiment, nested within a broader contracting approach—[33]), Haiti, Afghanistan and Liberia. In these countries, various models of contracting arrangements started respectively in 1997, 1999, 2003 and 2009, which is—in most cases—earlier than the beginning of PBF implementation, which was introduced in Rwanda in 2002, DR Congo in 2005, Burundi, Cameroon and Côte d’Ivoire in 2006. This suggests there may have been an evolution in supported models from ‘contracting’ to PBF.

In terms of type of programme implemented, the analysis shows that the PBF arrangements that are most commonly in place across FCAS are those described in the World Bank’s *PBF Toolkit* (perhaps unsurprisingly, since the World Bank is often the funder of PBF programmes, as detailed below). The vast majority of the programmes analysed cover a comprehensive list of indicators, usually related to the basic and complementary health service package that facilities are supposed to provide, with a focus on maternal and child health. However, a few programmes, in particular early and small-scale ones had a disease-specific focus. This is the case, for example, for the early pilots implemented with PEPFAR funding in Côte d’Ivoire since 2006 [34]. S2 Table provides further information on the PBF programmes analysed, their features and timing of implementation.

### Exploring patterns of PBF adoption

Following the framework proposed by Witter et al. [1], we looked first at PBF adoption and policy formulation. In this section, we attempt to find patterns across settings and to link them to the initial hypotheses on FCAS settings. It is important to stress that those hypotheses are not mutually exclusive; multiple hypotheses can hold at the same time in a same country.

We found that only a minority of reviewed documents justifies the introduction of PBF schemes in relation to the specific country settings, and these do so primarily by flagging the low achievements of the country in terms of health coverage, quality of care, or efficiency of services—or more often a combination of these reasons. These, broad ones are often the only mentions of contextual elements shaping PBF and its adoption. The logical link between this situation and the introduction of PBF—that is, the theory of change with respect to the country’s situation—is frequently left quite loose, especially in documents produced in recent years. Most of the early documents, those written in the 2000s, allude to the idea of extrinsic motivation of health workers and the need to rebuild the local health system (which, they argue, can be done through PBF). The documents introducing and commenting on the early schemes in Rwanda and Burundi [35,36] underline that input-based approaches did not seem to produce good results in a short time-frame and, therefore, output-based models ought to be given a chance. The link between experiences of conflict and fragility and the emergence of PBF is almost never explicit in the documents we reviewed, the main exception being early Rwanda PBF documents [37].

In addition, in order to explore how contextual elements are considered when discussing PBF adoption, we conducted an analysis of the PBF Community of Practice using the key-word ‘context’, and we noted that this too led to few results. A few members highlighted, in the Google-group discussions around 2010–2012, that ‘context’ was key but never really explained what element of context mattered and how. A discussion of the first systematic review of PBF (the 2012 Cochrane review—[13]) renewed this debate, with people noting that most studies have a quantitative bias that leads them to overlook contextual factors and the role they play. On the Google group, the last substantial discussion of context took place in 2014 and the message at that point was different, saying that “context should not lead us to alter the principles of PBF” and that “PBF can also make the context more positive”.

We then interrogated the literature to test our initial hypotheses. The first set of hypotheses (1–3) is not linked to fragility *per se* but to the **larger-than-usual place external actors often have in FCAS**. Indeed, it appeared that a key element to drive the adoption of PBF has been the presence and influence of external actors—donors, international organisations and NGOs—that are able to convince the government to implement, or let them implement, pilot or more fully fledged PBF schemes that they see as the best way to making rapid progress in improving health-care services and realising health development plans (e.g. in the Republic of Congo and Burundi). In recent years, the rhetoric of these external donors as reflected in the documents reviewed has particularly relied on PBF as the best way to achieve universal health coverage (e.g., [38]).

The literature review did reveal a very clear presence of such external actors at the onset and throughout the implementation of PBF schemes, with some very visible actors such as the NGO Cordaid (Caritas Netherlands) and the World Bank. It is possible to discern two main patterns of interaction between those external actors and local governments: (1) a piecemeal approach whereby PBF is first implemented in a pilot area with little government’s involvement and then, sometimes after a few other pilots, scaled up to large portions or the entire country; and (2) a full-scale approach in which the external actor strategically influences the set-up of a large scale or nationwide scheme, with the government’s close involvement. The first scenario is more common in countries where PBF started early, before 2007–2010. The list of countries includes Central African Republic (CAR), Côte d’Ivoire, Mali, Cameroon, Burundi, Lao PDR, Rwanda, DR Congo, and the Gambia and Tajikistan (two exceptions in terms of timing as their schemes started in 2012). The rationale for the piecemeal approach, as found in the documents, seems to be for the external actor to test PBF but also to introduce national actors to the concepts and working of PBF, and to showcase and promote the idea. The version of PBF that is introduced through those pilots can be relatively independent from the health authorities (e.g. CAR—[39]), with the organisation piloting PBF creating the purchasing agency and carrying out performance verification. Even in cases where good collaboration with health authorities is claimed (e.g. Burundi—[40]), early pilots tend to operate outside of government frameworks. Ad-hoc PBF policies are typically drafted *after* the pilot projects, immediately before the introduction of a larger project or the PBF scale-up (e.g., Côte d’Ivoire, Comoros—[41,42]). Whether such policies reflect genuine changes in strategy from the Ministry of Health (MoH), independently of proposed funding, is not possible to tell from our sources.

It must be stressed that the documents that were reviewed and that were part of the effort to ‘sell’ the PBF model rarely provided very rigorous evidence of the impact of PBF: causal frameworks are usually weak, possibly in part because evaluations, when they exist, have often been designed well after a pilot has started (preventing the use of more rigorous evaluation methods such as, for instance, randomised control trial or sometimes even difference-in-difference). Most documents provide anecdotal evidence, which is usually presented for what it is, but has, nevertheless, been used to substantiate success story narratives. Rwanda is an exception: it had an early, rigorous, impact evaluation supported by the World Bank [8,35]. Most of the PBF pilots reviewed were succeeded by a larger project, often involving the same external actor(s), and the documents related to this new project inevitably mentioned the encouraging pilot scheme as a key element for the new project. For example, Gautier mentions that in Mali the *apparent* results of the PBF pilot generated a strong engagement of national authorities and led to its inclusion in the strategic plan for the health sector ([43]–emphasis added), while in Burundi there is mention of the good adhesion of the different actors [10]. In one case, Sierra Leone, the pattern was reversed: an NGO small-scale pilot took place after the national project had been on-going for a few years. The objective of the pilot was, however, the same: to demonstrate that PBF could work well in a context were the national level PBF was not considered as working particularly well [44]. Whether this showcasing strategy is, in fact, effective and required is not possible to say; it may be that the external influence was such that PBF did not need to be ‘sold’ at all. In DR Congo, documents are explicit about the role of donors pushing for and funding PBF (e.g. [45]).

The second pattern consists in the immediate launch of larger projects, with donor funding, in almost all cases from the World Bank, and with the involvement of the local Ministry of Health. This was the case for Djibouti, Nigeria, Sierra Leone, Chad, Zimbabwe, Liberia, and the Republic of Congo. In many of those countries, the introduction of PBF happened slightly later than in the first set of countries. It should be added that the ‘showcasing strategy’ may have applied here too, with study tours and workshops being arranged to countries where PBF was already up and running, such as Burundi, Rwanda and the DR Congo, as well as Cameroon [46]. Such international tours and cross-country references are not limited to cases of the full-scale approach: Burundi’s PBF documents are full of references to Rwanda that add to domestic success stories (e.g. [47]).

These two sequences and the top-down, externally-driven, processes are probably not FCAS-specific. A recent study on Tanzania concludes that “the P4P policy process was highly political with external actors playing a significant role in influencing the agenda in Tanzania, leaving less space for the Government of Tanzania to provide leadership in the process. Norway in particular, took a leading role in setting the agenda” (p. 1) [48]. Other examples of the key role played by external actors include Mozambique and Kenya [49]. The hypothesis, though, is that the imbalance is even stronger in FCAS, which have less capacity or financial possibility to critically assess or modify projects from external partners. It may be further accentuated by the weaker policy and regulatory frameworks of FCAS, as described below. This may explain why most of the early PBF experiences were in FCAS.

**Fig 2:**
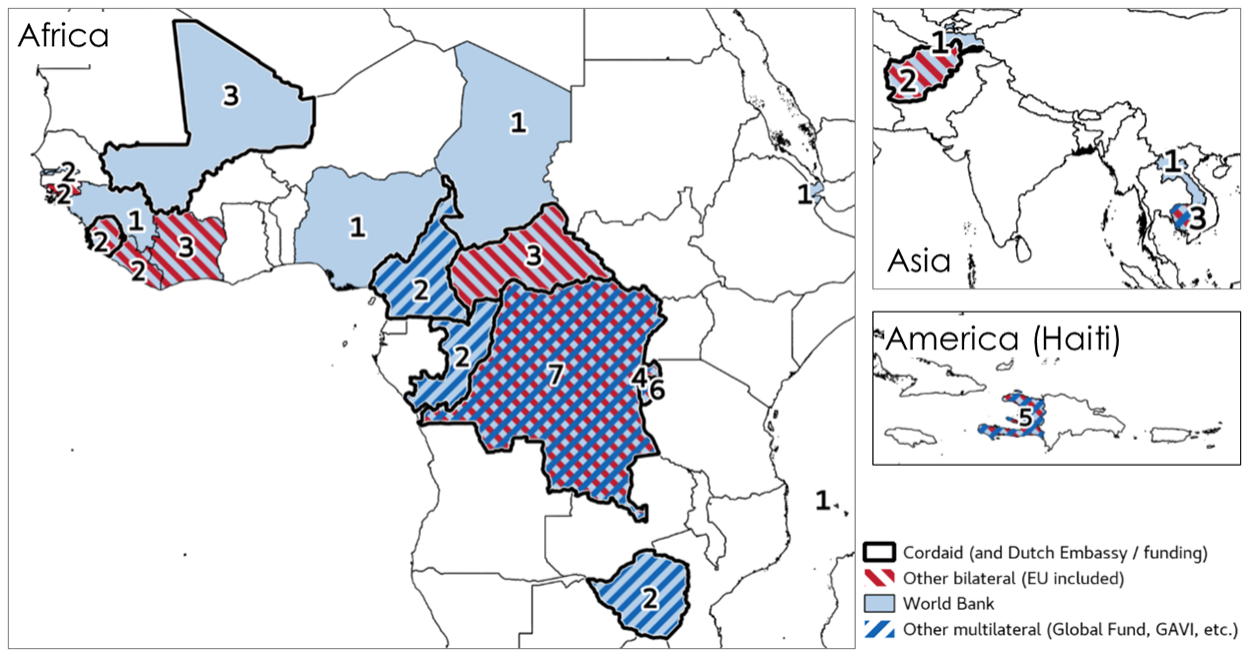
Diffusion of PBF, by external funder. Note: figures indicate the number of different PBF schemes implemented in 2002–2016. Source: compiled by the authors using the documents presented in S1 Table (in particular programme documents).

**Fig 3:**
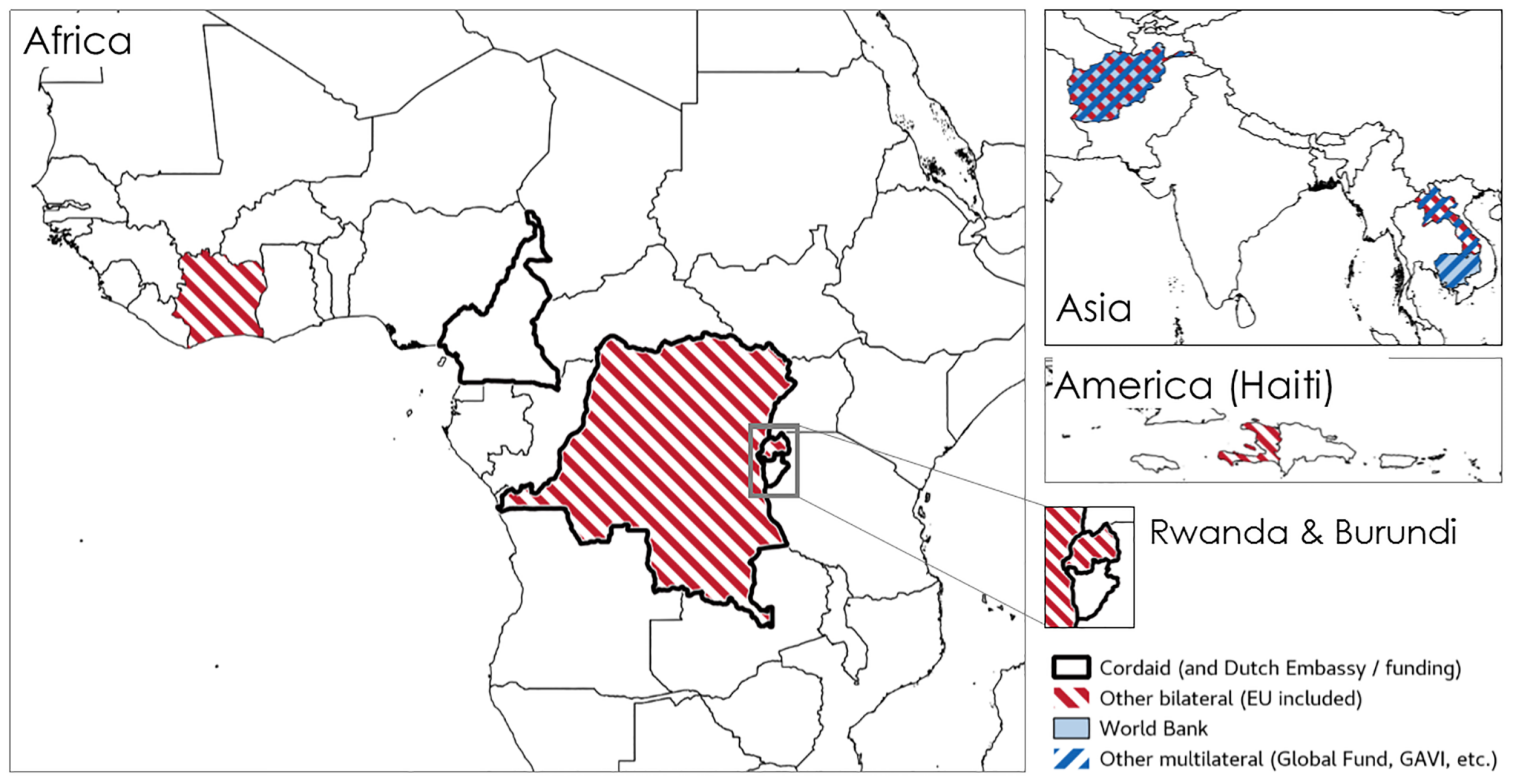
Diffusion of PBF, schemes by funder (2007). Source: compiled by the authors using the documents presented in S1 Table (in particular programme documents).

**Fig 4:**
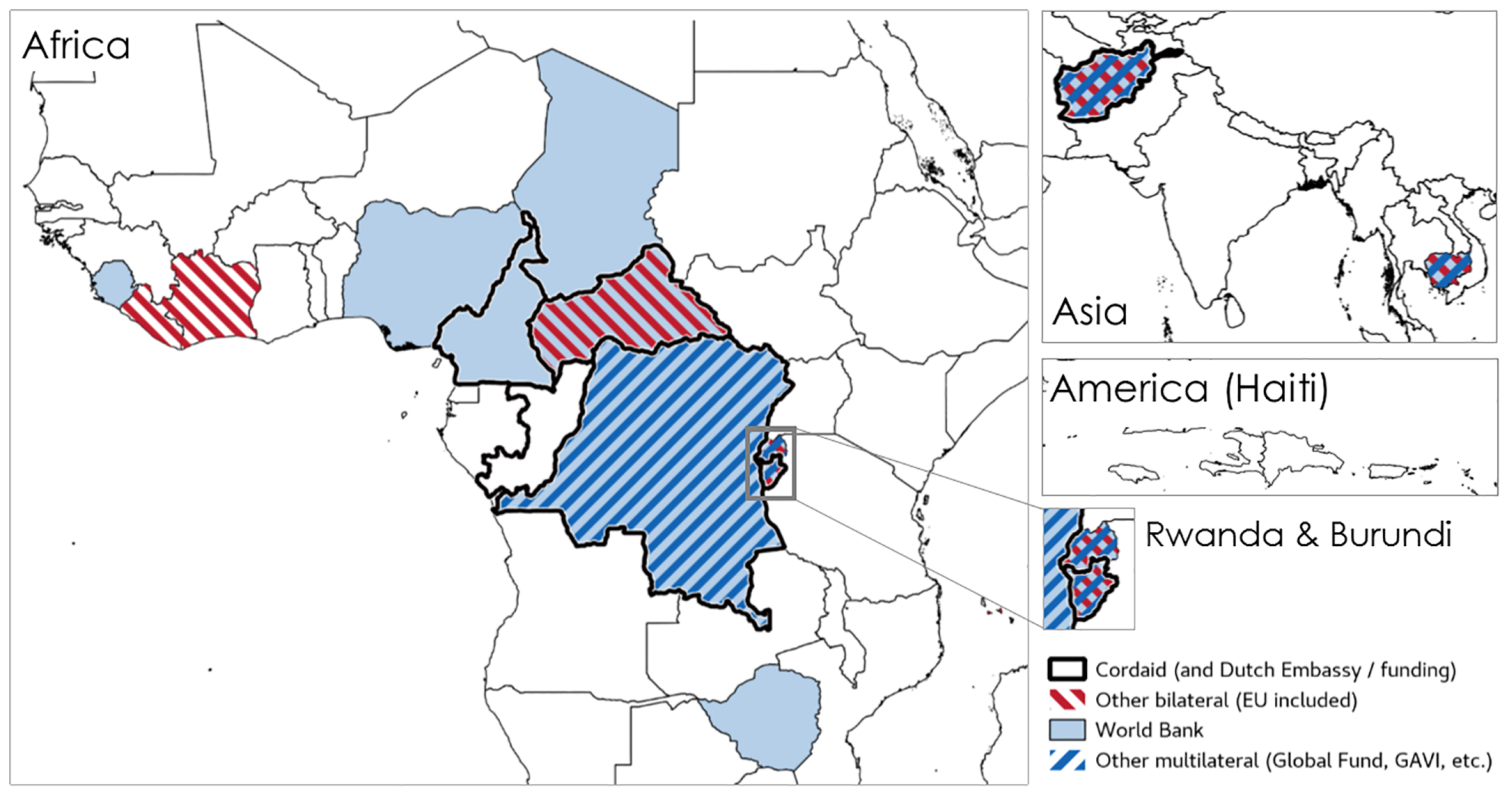
Diffusion of PBF, schemes by funder (2012). Source: compiled by the authors using the documents presented in S1 Table (in particular programme documents).

**Fig 5:**
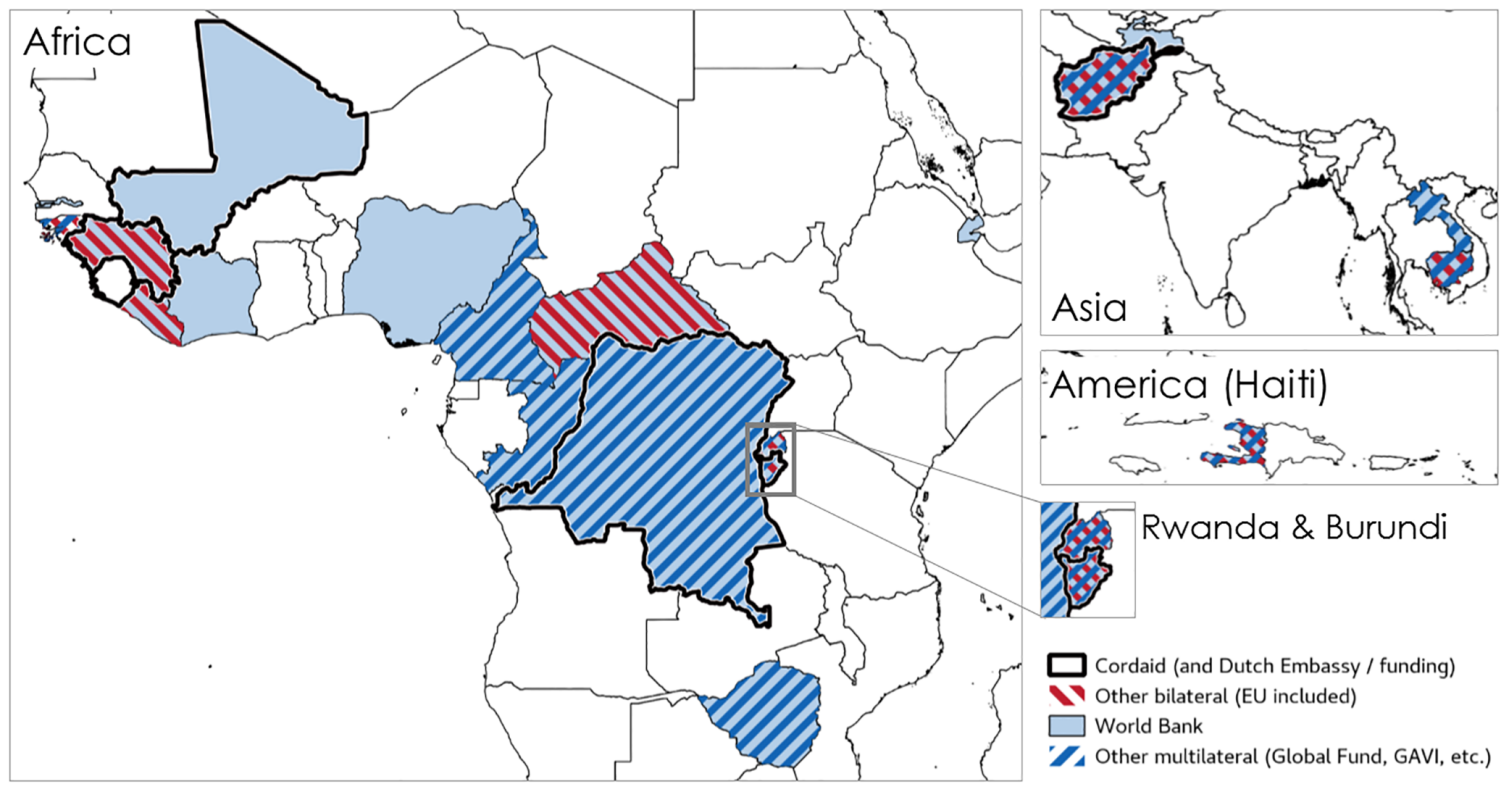
Diffusion of PBF, schemes by funder (2017). Source: compiled by the authors using the documents presented in S1 Table (in particular programme documents).

It is also interesting to map the role of a small number of external actors in the diffusion of PBF geographically and other time. The series of maps presented below shows how PBF spread to FCAS in the past the 10 years (Figs 2 to 5). Some funders, in particular Cordaid, USAID (for example, in Liberia) and the European Union (DR Congo) experimented with the first schemes. In some cases, schemes co-existed and, over the past ten years, countries like DR Congo and Burundi have seen respectively seven and six PBF schemes implemented on their territory as new funders and implementers arrived (Fig 2). In the more recent years (especially since 2012), as the approach become more popular and more visible (it was, for instance, very visibly featured in institutional website, social media, and international conferences), the World Bank and multilateral donors (UNICEF, GAVI, Global Fund, etc.) have become key players (Figs 4 and 5). Actors like Cordaid largely contributed to promoting the approach through conferences and communication campaigns. They were also venturing into a number of new countries (Figs 4 and 5).

**Fig 6:**
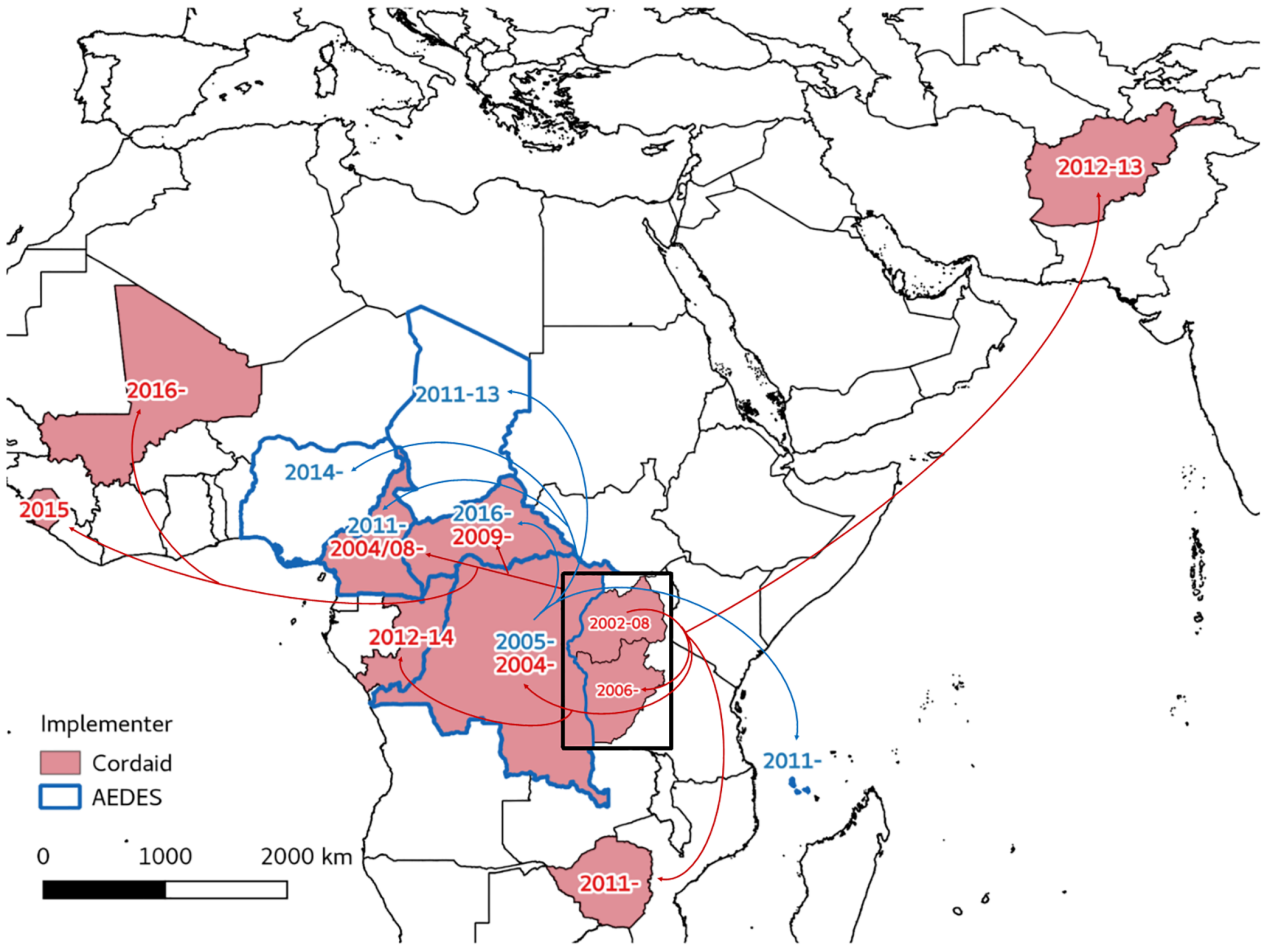
Diffusion of PBF (Cordaid and AEDES). Note: arrows indicate the next country where the organisation implemented PBF. Source: compiled by the authors using the documents presented in S1 Table and information available on the websites of AEDES, Cordaid, and SINA Health.

A pattern in the adoption of PBF which follows influential external individuals and institutions was also noted (Fig 6). Although not directly discussed in the documents we reviewed, the authorship and acknowledgements reveal a geographic pattern in the diffusion of PBF, across the Great Lakes and initially markedly in French-speaking countries, in which (external) individuals (Soeters, Fritsche, Meessen, to name but a few) and organizations (Cordaid, AEDES, World Bank) played a key role. For example, the presence of AEDES’ technical assistant, who had been previously working in the (EU-funded) PBF scheme in DR Congo was important to introduce the concept in Comoros (personal communication). One study links Cordaid interest for, and implementation of, performance-based approaches to the changes in the organization of the Dutch bilateral development cooperation and the reorientation towards results [50]. Mapping the diffusion of PBF across FCAS by Cordaid and AEDES, we find a set of initial experiences in central Africa -Rwanda, DR Congo (South Kivu) and Burundi for Cordaid and DR Congo for AEDES. It is in those countries that the two organisations developed their expertise on PBF in fragile settings. The creation of a community of practice early in the process [51] and the development of study tours has probably also contributed to anchoring the central African experiences as references. It must be noted that the maps below do not include non-FCAS countries, although experiences of these individuals and organisations in non-FCAS countries have, of course, also influenced the diffusion of PBF in FCAS countries.

Our fourth and fifth hypothesis were linked to the type of societies FCAS are and the sort of government they have: they typically are societies with a very **low level of (interpersonal) trust, whose governments have low capacity,** in particular to manage funds in an appropriate manner. PBF may appear as providential with respect to both issues: it substitutes difficult (interpersonal) relations with clear-cut contracts and sets clear financial procedures, often sending external agents to manage funds.

Few documents explicitly note that the lack of trust between levels of the health system and widespread corruption and lack of accountability were influential in the adoption of PBF (hypothesis 4). Between the lines, though, there are acknowledgments that PBF fits well in the agendas of the societies that are being rebuilt. Toonen et al. [32] explain that the entry point for PBF in Mali was “lack of trust between these actors [i.e., actors at different levels of health systems] and confusion about the distribution of their roles and responsibilities” (p. 43) at decentralized levels, as “all felt responsible for health services at decentralized level” (p. 38). They also argue [32]that PBF in Mali may ultimately contribute to improving this situation of mistrust because a new framework (PBF) is established for collaboration. The situation is similar in other countries: in Burundi, a country deeply affected by low interpersonal trust, the concept of PBF faced very little resistance within the MoH and was presented as part of the new good governance package, beyond the fault lines inherited from the past [52]. Burundi is far from being the only country where PBF echoes aspirations (or at least a political discourse of) accountability: a report from Cameroon highlights that one of the elements that contributed to putting PBF on the MoH’s agenda was that it was in line with the political discourse toward more accountability and the Presidential priority of the fight against corruption [53]. PBF however does not seek to address the root of mistrust between local actors.

The **lack of trust can also be between donors and the government** (hypothesis 5), and is not specific to PBF or to FCAS. Few documents explicitly discuss this issue but in Liberia, Sondorp and Coolen [54] found that “USAID opted to set up a programme, close to but essentially separate from government. [… It] intended to provide a mechanism which gives the government more leadership but with a number of checks and balances in actual disbursements” (p. 24).

In particular, fiduciary concerns and the perceived lack of capacity of the government to manage external funds provide one of the possible rationales for implementing PBF schemes that introduce a purchasing function clearly detached from the government. This may take the form of a new non-governmental agency funded by international aid that persist years after the schemes have started (e.g. DR Congo, with the creation of Purchasing Agencies in the form of the *Etablissements d’Utilité Publique*, EUP, or the *Agences d’Achat de Performance*, AAP, the latter being also created in NGO-run PBF projects in other countries). In other places, the role is given to newly created ‘PBF project implementation units’. In this case, the implementation unit is officially embedded within government, but in fact, follows the pattern of an independent service authority [55] with its staff paid by international aid and the entity largely ‘preserved’ or isolated from government. The motivation for such mechanism appears to come from both government and donors. The Côte d’Ivoire’s World Bank PAD specifies that “the government of Côte d’Ivoire requested to use a ring-fenced financing mechanism for the fiduciary aspects of the project” (p. 40) [56]. Sometimes, as in the case of Zimbabwe, the country simply cannot be directly engaged by the donor (the World Bank, in this case) as it is in arrears. An external party, Cordaid was then chosen by the donor to play this role [57]. Zimbabwe and Côte d’Ivoire remain exceptions, not in the sense that there is a ring-fenced mechanism, but rather in the sense that documents explicitly acknowledge that this arrangement is motivated by state fragility. Such arrangements where the purchasing agencies are external to the government are always described as transitory.

It is interesting to note that non-FCAS countries, such as Benin and Senegal, also have project implementation units and external implementing agencies. We were unable to assess whether FCAS country are substantially more likely to have such mechanisms or whether this is simply countries following a ‘trend’ established in some of the early PBF countries. We found that project implementation units and external implementing agencies are not systematically associated to a particular donor either. What some FCAS countries such as the DR Congo seem to exemplify, though, is that the state of fragility—namely, the recognized lack of financial management capacity of the government—makes it possible to implement complex schemes where important functions (purchasing and implementation) remain outside government for a long time. They are deemed, possibly rightfully so, too complicated for the state to manage [58]. This also means that, in those cases, PBF may not be doing much to reinforce the capacities of the state (a key part of its fragility).

Our sixth hypothesis was linked to the socio-economic and political distortions engendered by conflict: ***de facto* decentralisation, and the flexibility of new institutions**, including *per diem* mentalities inherited from humanitarian interventions. All might make FCAS countries less resistant to PBF-types of innovations. However, the hypothesis that PBF finds a more fertile ground in FCAS because they are more decentralised and therefore less prone to resistance did not find much echo in the documents. A reason may be in the way we had framed this hypothesis in the beginning: the sort of decentralisation we had in mind is a *de facto* decentralisation with health facilities and health district having become (in actual practice, rather than formally) more autonomous in their management because of periods of war during which the state struggled to ensure direct contact with and control over them. However, because of the informal nature of such decentralisation, an explicit discussion of it is unlikely in the documents, and was rarely found. Only in one of the most extreme cases, the DR Congo, it was hinted that the situation of de facto decentralisation may favour the introduction of PBF as fewer battles have to be fought at the central level and local agents may be more used to dealing with external parties [59,60]. However, what a series of documents (including on the DR Congo) have tended to highlight is how the lack of formal decentralisation, or botched decentralisation processes, have in fact hindered the development of PBF because competences and responsibilities are unclear or disputed between different health authorities.

An issue that is well-mentioned, though, is the weakness, and the consequent flexibility, of existing institutions and the room for institutional change that characterizes FCAS settings, especially in the immediate post-conflict period. Toonen and colleagues [32], discussing the case of the introduction of PBF pilots in Rwanda, points out that the country “was faced with a post-war situation in which institutions and governance structures were practically non-existent. So setting up new (PBF) institutions where an NGO acted as the purchaser (the AAP–*Agence d’Achat de Performance*) did not pose too much of a problem. In Mali and Ghana, the healthcare sectors do have governance institutions and ‘rules of the game.’ This provides different constraints, and different opportunities. […] Particularly in those countries with a well-established health architecture, such as most West African countries, a contracting approach like RBF needs to be embedded in the prevailing national governance structures” (p. 9). Indeed, in some of the countries considered, such as Burundi, Rwanda and Côte d’Ivoire, PBF was adopted and ‘codified’ during the post-conflict reorganization of national policies and strategies [36,41,61].

Related to this issue is a more specific hypothesis (hypothesis 7) that **interests and power relations may be less entrenched in FCAS** and, therefore, they are less likely to resist PBF or push for its ‘indigenization’. There are few documents that refer to this, but Bertone et al. [62] make the case that in Sierra Leone, professional groups were less powerful and routines less established after the war, which made them less likely to unite against health system reforms. This may not be the case of all FCAS countries. Countries such as Comoros, Mali, Nigeria and Cameroon suffered a conflict that is (or was) less damaging or affected only a portion of the country. In those cases pre-existing formal and informal institutions may be stronger and in a better position to shape the way PBF is implemented and its potential effectiveness.

### Exploring patterns of design and adaption

In relation to our expectation that there might be **more variation and adaptation in PBF models in FCAS** (hypothesis 8), our analysis of the existing documentation shows that, after its early development notably in Rwanda, PBF has subsequently been designed and implemented in very similar, almost identical ways in most of the settings. The only notable exception concerns the pragmatic adaptations to PBF made at implementation stage when the programme faced unexpected contextual events, such as the Ebola epidemic in West Africa, recrudescence of armed conflict in Nigeria and CAR, or influx of refugees from other regions in Cameroon. These adaptations were related less to capacity issues than the crisis-prone contexts. In Sierra Leone, the national PBF project kept providing services during the Ebola outbreak, although it was decided to discontinue the verification procedures due to the impossibility and risks of travels [44]. In Guinea, a new PBF project is being adapted to cover system functions relevant to the epidemic, such as notification and confirmation of Ebola cases, contact searching, and appropriate burial measures [63]. In northern Nigeria, during the humanitarian crisis caused by Boko Haram’s insurgency, PBF contracts continued with the clinics which were still functioning, providing funds for facilities and incentives for health workers. At the same time, in districts where IDP camps were set, camp clinics were established and sub-contracted by nearby PBF primary contract holders. In heavily affected districts mobile clinics were set up, based in nearby safe areas and contracted to carry out a ‘hit and run’ strategy which consisted in entering into insurgency affected areas following security clearance to provide health services.

Following the 2013–2014 humanitarian crisis in CAR and Cameroon, the existing PBF programmes were adapted to increase the proportion of population to be exempted from fees. In CAR, while under normal conditions facilities are allowed to provide services for free to the very poor for a maximum of 20% of the services provided (these services are paid 4 times more than the corresponding indicator for non-poor), the exempted population can increase to 50% or 100% of patients in case of humanitarian crisis [64]. Similarly, in the eastern regions of Cameroon with a high number of refugees from CAR, the (already existing) extra payment to cover for free services provided to the very poor, which was capped at 10%, was increased to 20% for the duration of the crisis [65]. Additionally, the current World Bank pilot in CAR (which started in early 2017) has been designed to respond to the early recovery conditions, including a “vulnerability score” in facilities’ bonus calculations, which provides higher payments to less secure areas, as well as a “quality improvement bonus”. This is a cash-based, non-performance-based bonus to be used to recruit new staff where facilities lack qualified personnel, rehabilitate infrastructure and purchase essential equipment [66]. Given the early stage of the PBF programme, it is not possible to assess the functioning and effectiveness of these mechanisms.

### Exploring patterns of implementation and health system effects

In relation to our ninth hypothesis on the **challenges of sustaining PBF in FCAS contexts**, comparatively less information was uncovered in the documents retrieved. In some cases, these are evidenced by the case of start-stop(-start) funding in some countries. The best documented case is that of Chad, where a recent study [46] stressed that, while external entrepreneurs (in that case, the World Bank) proved sufficient for a ‘superficial’ adoption of PBF and its introduction as pilot, the external influence was not enough to sustain the project, which was discontinued 20 months later. Discussions are now on-going in Chad for a possible new start of the programme (personal communication). A similar case of start-stop approach, followed by discussion about a possible re-introduction of PBF, in Sierra Leone has not yet been fully documented. However, other cases in non-FCAS countries and areas (such as Benin or southern Mali—[67]) point to the fact that start-stop approaches and lack of sustainability are not specific to fragile settings. Overall, it appears that, in countries where PBF is introduced as part of a wider set of health system and health financing reforms, based on a results-orientation, PBF may be more likely to be sustained. Some FCAS countries, such as Rwanda and Burundi, provide positive examples of this at least.

In relation to our expectation of **variable PBF health system effects in FCAS** (hypothesis 10), there are limitations in the literature in terms of how far health system effects are reported, as well as the difficulty of attribution of changes to PBF. We found very limited evidence of, or explicit reference to, contextual interaction with direct as well as indirect apparent effects of PBF on governance, financing, human resources and drugs supplies for some of the FCAS countries reviewed.

#### Governance and health information

It has been argued that PBF could act as a tool to reinforce the overall governance of the health system [7,68]. Little empirical evidence exists so far, in particular concerning how elements of the context can affect the influence of PBF on governance. Among the studies reviewed, Remme et al. [39] stress that PBF did reinforce certain elements of good governance in a pilot in CAR, especially at provider level, and so did the process evaluation in Zimbabwe [69]. In Haiti, the introduction of PBF forced the government to beef up the State’s health data reporting system for validation of activities performed; however the 2016 Hurricane Matthew appears to have halted this process [70].

As with other pillars discussed below, it is likely that different types of fragility (e.g. humanitarian/ political crisis, early recovery, transition, or chronic fragility) would affect the relation between PBF and governance in different ways, and also that the levels of the system would also be differentially affected. For example, PBF could reinforce governance at local level (e.g. that of the providers towards patients and communities), especially in extremely fragile contexts where the state or national authorities play a marginal role in service provision and regulation, but could have a much less significant role in terms of reinforcing governance and stewardship of higher levels, especially when the PBF adoption process is externally driven, and where ownership and capacity for implementation by local authorities is low [39,46,71].

#### Health financing

A synergy has been noted between PBF and user fee removal (for example in Burundi—[10]), with the former replacing facility income lost through the latter. While this is not specific to FCAS, there is an even stronger case for removing fees for essential services during crises, and in countries or sub-regions affected by conflict (such as northern Mali—[72]), this has been a rationale for PBF introduction. However, this linkage is also problematic if the funding for PBF is limited and there is an expectation that PBF will incentivize and fund quality improvements; clearly if it is merely substituting for user fees in an under-funded system, then quality improvements cannot realistically be expected [73].

The relationship between PBF payments and overall facility income will be important to its effects in all settings, and in many FCAS, such as Chad, PBF is the only financing source for providers along with user fees [74]. Whether this is more common in FCAS is yet to be established though.

#### Human resources for health

As for facilities, similarly for individual health workers, it is possible that PBF bonus may represent a higher proportion of their income in FCAS where salaries are (often) lower and less regularly paid and there may be fewer opportunities for highly-paid private practice. Some evidence exists on the proportion of PBF within the income of health workers for Sierra Leone, where PBF represents about 16% of the salary and 10% of total income for primary health workers in rural facilities [75] and Chad. In Chad, evidence is based on an example of only two health workers: PBF represented respectively 79% and 45% of salary for a vaccinator and nursing aide [74]. In contrast, the average bonus payment per month for nursing staff is approximately 10% of their average monthly salary in Tanzania [76].

Another issue concerns how the individual PBF bonus was shared within the facility, where a tension between a focus on performance and cultural expectations of solidarity may exist. One of the documents reviewed, focusing on Chad, describes how facilities took the decision to share the bonus not on the basis of individual performance, as it should be done according to Chad’s PBF implementation manual, but based on cadre and seniority (which is the approach preferred in most PBF schemes) in order to reduce tensions and ensure solidarity [74]. In Sierra Leone, a study of PBF [75] found both instances of solidarity (i.e., sharing bonus also with non-eligible staff to ensure that all workers receive an income) and tensions between health workers because of the distribution was perceived as unfair, or because of misappropriation of bonuses. It is likely that this issue is not related to FCAS status (see for Tanzania—[76]), but rather to specific cultural and social values in each setting. Two studies from Nigeria [77,78] discuss how a number of contextual and implementation factors affect the motivation of health workers and the effectiveness of PBF implementation. These factors included: uncertainty in earnings due to delays in payment, lack of communication, and lack of knowledge concerning the tool used to assess individual performance; lack of understanding of the P4P scheme; variations in the role of health facility managers; and differences in infrastructure availability between facilities.

#### Drugs and infrastructure

Not much information was found on the effect of PBF programmes on the drugs and pharmaceutical regulation pillar. Because of security issues, absence of a formal private retailing sector at the peripheral level, and the less-than-consolidated distribution lines, health programmes’ pharmaceutical procurement and distribution functions in FCAS are often centralized. Even in those PBF programmes guaranteeing local financial autonomy and access to banks (as in Chad and CAR), local managers were reported to depend on the state’s systems for drugs provision.

Beyond the capacity to adapt in acute humanitarian situations described above, the documents reviewed seem to show that PBF mechanisms are less able to address the issue of rehabilitation and equipment of facilities in recovery settings, unless coupled with extra, non performance-based funding. As a result, a number of cash or in-kind bonuses have been introduced in PBF programmes (e.g. Adamawa State in Nigeria, CAR, Côte d’Ivoire, Haiti—[56,66,79,80]). Though this problem may be starker in FCAS settings, a study in Tanzania (non-FCAS) noted a similar point. It stressed that it is important to consider contextual issues when implementing PBF schemes in low income settings, and highlighted the importance of basic infrastructure and staff being available before implementing the scheme, as these constraints are beyond the control of providers and managers [81].

## Discussion

This review of the published and (largely) unpublished literature has examined a neglected area: the effect of context on the adoption, adaption, implementation and health system effects of PBF, focused on FCAS settings where many of the PBF programmes have been implemented. Our findings, still tentative in the absence of more evidence, do suggest that PBF has been commonly adopted in FCAS settings, and also earlier on in the history of the expansion of PBF. PBF in these contexts appears to have followed in the footsteps of contracting, which was previously the favoured model for donors supporting service delivery in FCAS settings.

Our hypotheses on why FCAS settings might offer more opportunities for PBF were largely supported by the documentary evidence, although it is clear that many of these elements are not exclusive to FCAS settings—though perhaps commonly more extreme in them. A few broad elements, such as lack of trust, both within the health and governance system but also between government and donors, emerged as important factors. How these elements are then affected by PBF—for example, whether trust is rebuilt or merely circumvented—is not yet clear.

Another factor which emerged is the fluidity of institutions and the influence of external actors (in particular, a limited number of key agencies). Indeed, going beyond PBF, this article is a study of how a ‘new’ idea in global health and international aid has spread and adapted, or not, to national and local environments. It complements the growing body of literature on the adoption, diffusion, and change of global norms, ideologies and ideas in international development and health [82–84]. In particular, the paper looks at the place and agency of national actors [85,86] in a context marked by conflict and the professionalization of international development [87].Our analysis of how the PBF concept was diffused by organisations and individuals across FCAS settings is not necessarily exclusive to PBF and may link to wider trends in aid dependent countries. It has been documented in health financing [88] and in international development more generally [87]. Working on the diffusion of innovations in health, Fitzgerald et al. [89] point out the important role of social mediation and intermediation, something clearly visible in the case of PBF and its travelling experts and community of practice. Additionally, our finding on the lack of governmental ownership and donors’ influence around PBF adoption echoes that of a recent study [90], which also includes Rwanda, Burundi, DR Congo; and our finding on the role of some individuals in the diffusion of PBF is in line with Shroff et al. [49]’s analysis of the scale-up of PBF programmes in 11 countries, including in some FCAS settings, which mentions the presence of ‘global health financing experts in the country’ as a key enabler for countries like Burundi, Rwanda, Cameroon and Mozambique. The influence of such experts and institutions is not peculiar to FCAS but, in line with the critical development studies literature [91] and in light of the flexibility of institutions and weaker position of local elites mentioned above, it seems likely that external experts and institutions are more influential in FCAS and that their legacy can stretch over a few countries.

Similarly, Barnes et al. [92] examine the emergence of the PBF concept, focusing on three, non-FCAS countries (Tanzania, South Africa and Zambia). They conclude that there is a degree of capture by external agencies, and that participation for Africans depends on position within government, relationships with funders and awareness of informal opportunities. They conclude that “a source of African agency lies in the strength of the country’s health system” (p. iii) [92], which, if true, has important implications for FCAS. Push-back on the PBF concept from South Africa is cited as an example. They identify clear commitment to PBF at international and national levels, despite weakness of the evidence, because of the ‘political capital’ it gives to those working to promote it. The authors see this as the main reason for the lack of critical engagement with the concept, normatively or practically.

In their analysis, Barnes at al. [92] also highlight the lack of appreciation of the context-specific nature of PBF and of the causes of its success or failure to date. Indeed, what emerges from our analysis is the fact that explicit justifications of the rationale for the adoption of PBF in relation to contextual needs are extremely limited and -it appears- little consideration is given in the documents we reviewed to the more granular specificities of the context and how they may affect adoption, adaption and design, implementation and effects of PBF. For example, the documents we reviewed provide almost no mention of the local contextual factors that may have influenced the take-off of PBF in Rwanda, one of the very first schemes and probably the most cited of all. However, the grey and academic literature on government in Rwanda has taken a great interest in the idea of *imihigo* [93–95], a traditional concept that can be understood as a ‘performance contract’ between an authority and citizens, which has been ‘modernised’ and institutionalised by the Rwandan government. Has *imihigo* provided a fertile ground for PBF in Rwanda? This is a possibility but it would need to be evidenced by further research. However, only one of the document we reviewed mentions *imihigo*, to stress the risk possibility of bias in PBF evaluation [96].

Contextual elements are likely to strongly affect the relevance of PBF adoption, and the lack of analysis of contextual needs and features hinders the adaptation of PBF design and the detailed assessment of the relationship between effects and local context. This may promote externally-favoured, rigid models [97]. Narratives of success of PBF in other settings are commonly cited in the policy adoption process, but whether these are potent or cover more direct persuasive factors such as the availability of funding from donors is uncertain. Some of the early PBF literature (for example, [27]) already noted the potential problem of transferring policy recommendations across different settings, highlighting that not just the need for strong political support but also that adaptive health systems, ability to absorb risk and strong information systems are required for the implementation of PBF. (The costs of monitoring are of course closely tied to the functionality and robustness of information systems). Contexts also influence the likely internal distribution of rewards within a scheme (e.g. rewarding facilities in more densely populated areas as against those with remote populations). The Cochrane review of 2012 [13] also highlighted the likely impact of different organisational settings, which was then under-studied. More recently, Renmans et al. [16] highlighted the importance of context, including the role of ideology and values in shaping PBF, while noting that studies do not provide in-depth analysis of how contexts affect implementation and results.

In relation to implementation, we do find a degree of adaptation to different FCAS contexts, particularly humanitarian settings, where the suitability of PBF as a financing model merits more exploration. It is harder, however, to identify patterns on sustainability and health system effects, partly because of the early stage of many programmes, but also because of the limited documentation and the varied settings. With a few patchy exceptions, the documents available lack a detailed assessment of health system effects and PBF is treated as a stand-alone funding mechanism rather than as an integrated health reform with impact on financing, but also human resources, health information systems, governance, drug supply, etc. Issues such as the integration of PBF within wider health financing modes are neglected. In this respect, more than answering, the review raises questions. For example, is there evidence that PBF is better adapted to specific types of FCAS settings (for example, early recovery rather than reconstruction, when more significant infrastructural investments might be needed)? How is it affected by different levels of pre-existing autonomy at facility level (e.g. some of the FCAS countries permitted facility financial management prior to PBF, which was relatively rare previously in more stable settings)? Can PBF be seen, is some contexts, as a continuation of the practice of humanitarian organisations in topping up salaries?

This review has clear limitations. Although we used a number of channels to try to obtain all relevant operational documents and reports on PBF programmes in FCAS countries, we will not have been able to find all. In particular, questions around factors driving adoption and political economy are sensitive and tend to be under-documented. To explore these in depth would require primary data collection through interviews and other qualitative methods, which is planned in the next stage of this work. We also recognise that the FCAS classification is easily challenged and very heterogeneous internally, as well as changing over time. Finally, the fact that we focused our search only on the literature concerning the health sector does not allow us to compare the use of (or lack thereof) performance approaches in other public sectors and address important questions pertaining to the reasons why PBF approaches started and seemed to find more fertile grounds in the health sector, and were only later, and so far more rarely, adopted in other sectors such as education. Rather than a review of grey and policy documents, interviews with policy-makers would probably help better understand those dynamics.

## Conclusion

While context is recognised to be important in a number of studies on PBF in low and middle income settings, this aspect has not been interrogated in any detail to date. Our analysis took a theory-led literature review approach and interrogated available published and grey literature to examine the relationship between FCAS contexts and the adoption, adaption, implementation and health system effects of PBF. We found that, within low and middle income countries, PBF has been more common in FCAS contexts, which were also more commonly early adopters. Very little explanation of the rationale for its adoption, in particular in relation with the contextual features, is given in programme documents. However, there are a number of factors which could explain this, including the greater role of external actors and donors, a greater openness to institutional reform, and lower levels of trust within the public system and between government and donors, all of which favour more contractual approaches. These suggest that rather than emerging despite fragility, conditions of fragility may favour the rapid emergence of PBF.

Overall, our analysis highlights the need for greater clarity on how PBF interacts with the contexts, and in particular with FCAS feature, both *ex ante* (at the stage of adoption and design/adaption) and *ex post* (during the implementation and the assessment of health system effects). A few, emerging adaptations of PBF, focused to humanitarian settings are documented, as well as some (limited) evidence of health system effects which may be contextually driven, but both of these areas require more in-depth analyses. They should also help explore a crucial question: the extent to which PBF is reinforcing—or not—fragile states. We also note the important role of early ‘reference cases’ and the prominence of certain implementers and funding organisations in spreading the PBF concept across FCAS (and non-FCAS) settings. Another area meriting more study is the political economy of PBF and its diffusion across contexts.

## Supporting information

S1 Table List of fragile and conflict-affected states included in the literature review. (DOCX)

S2 Table PBF experiences in FCAS, by characteristics, funder and stage of development. (DOCX)

## References

[1] Witter S, Toonen J, Meessen B, Kagubare J, Fritsche G, Vaughan K. Performance-based financing as a health system reform: mapping the key dimensions for monitoring and evaluation. BMC Health Serv Res. 2013;13: 367 doi: 10.1186/1472-6963-13-367 24073625 10.1186/1472-6963-13-367 PMC3849795

[2] Musgrove P. Financial and Other Rewards for Good Performance or Results: A Guided Tour of Concepts and Terms and a Short Glossary [Internet]. Washington, DC: World Bank Background Brief; 2011. www.rbfhealth.org

[3] Fritsche G, Soeters R, Meessen B. Performance-Based Financing Toolkit. Washington, DC: World Bank; 2014.

[4] Sekabaraga C, Diop F, Soucat A. Can innovative health financing policies increase access to MDG-related services? Evidence from Rwanda. Health Policy Plan. 2011;26: ii52–ii62. doi: 10.1093/heapol/czr070 22027920 10.1093/heapol/czr070

[5] Josephson E, Gergen J, Coe M, Ski S, Madhavan S, Bauhoff S. How do performance-based financing programmes measure quality of care? A descriptive analysis of 68 quality checklists from 28 low- and middle-income countries. Heal Policy Plan (Advanced Access). 2017; doi: 10.1093/heapol/czx053 28549142 10.1093/heapol/czx053 PMC5886109

[6] Soucat A, Dale E, Mathauer I, Kutzin J. Pay-for-Performance Debate: Not Seeing the Forest for the Trees. Heal Syst Reform. 2017;3: 74–79. doi: 10.1080/23288604.2017.1302902 10.1080/23288604.2017.1302902 31514675

[7] Meessen B, Soucat A, Sekabaraga C. Performance-based financing: just a donor fad or a catalyst towards comprehensive health-care reform? Bull World Health Organ. 2011;89: 153–6. doi: 10.2471/BLT.10.077339 21346927 10.2471/BLT.10.077339 PMC3040374

[8] Basinga P, Gertler PJ, Binagwaho A, Soucat AL, Sturdy J, Vermeersch CM. Effect on maternal and child health services in Rwanda of payment to primary health-care providers for performance: an impact evaluation. Lancet. 2011;377: 1421–1428. doi: 10.1016/S0140-6736(11)60177-3 21515164 10.1016/S0140-6736(11)60177-3

[9] Bonfrer I, Van de Poel E, Van Doorslaer E. The effects of performance incentives on the utilization and quality of maternal and child care in Burundi. Soc Sci Med. 2014;123: 96–104. doi: 10.1016/j.socscimed.2014.11.004 25462610 10.1016/j.socscimed.2014.11.004

[10] Falisse J-B, Ndayishimiye J, Kamenyero V, Bossuyt M. Performance-based financing in the context of selective free health-care: an evaluation of its effects on the use of primary health-care services in Burundi using routine data. Health Policy Plan. 2014;30: 1251–1260. doi: 10.1093/heapol/czu132 25533992 10.1093/heapol/czu132

[11] Binyaruka P, Patouillard E, Powell-Jackson T, Greco G, Maestad O, Borghi J. Effect of Paying for Performance on Utilisation, Quality, and User Costs of Health Services in Tanzania: A Controlled Before and After Study. PLoS One. 2015;10: 8. 10.1371/journal.pone.0135013 PMC4552688 26317510

[12] Huillery E, Seban J. Financial Incentives are Counterproductive in Non-Profit Sectors: Evidence from a Health Experiment. Paris: Science Po, Department of Economics—Working Paper; 2015.

[13] Witter S, Fretheim A, Kessy F, Lindahl A. Paying for performance to improve the delivery of health interventions in low- and middle-income countries (Review). Cochrane Collab. 2012; 10.1002/14651858.CD007899.pub2 22336833

[14] Blacklock C, MacPepple E, Kunutsor S, Witter S. Paying for Performance to Improve the Delivery and Uptake of Family Planning in Low and Middle Income Countries: A Systematic Review. Stud Fam Plann. 2016; Advanced Access. 10.1111/sifp.12001 PMC5434945 27859313

[15] Ssengooba F, McPake B, Palmer N. Why performance-based contracting failed in Uganda—An “open-box” evaluation of a complex health system intervention. Soc Sci Med. 2012;75: 377–83. doi: 10.1016/j.socscimed.2012.02.050 22560799 10.1016/j.socscimed.2012.02.050

[16] Renmans D, Holvoet N, Orach CG, Criel B. Opening the “black box” of performance-based financing in low- and lower middle-income countries: a review of the literature. Health Policy Plan. 2016;31: 1297–1309. doi: 10.1093/heapol/czw045 27126200 10.1093/heapol/czw045

[17] Paul E, Renmans D. Performance-based financing in the heath sector in low- and middle-income countries: Is there anything whereof it may be said, see, this is new? Int J Health Plann Manage. 2017;Advanced A. 10.1002/hpm.2409 28382750

[18] Renmans D, Holvoet N, Criel B, Meessen B. Performance-Based Financing: the same is different. Health Policy Plan. 2017; 1–9. https://doi.org/10.1093/heapol/czx030 28369426 10.1093/heapol/czx030

[19] Antony M, Bertone MP, Barthes O. Exploring implementation practices in results-based financing: the case of the verification in Benin. BMC Health Serv Res. 2017;17: 204 doi: 10.1186/s12913-017-2148-9 28288637 10.1186/s12913-017-2148-9 PMC5348780

[20] Cataldo F, Kielmann K. Qualitative research to enhance the evaluation of results-based financing programmes: the promise and the reality. HNP Discuss Pap World Bank. 2016;

[21] World Bank. Learning Agenda for Results-Based Financing in the Health Sector: The Health Results Innovation Trust Fund Learning Strategy. Washington, DC: World Bank; 2016.

[22] Martinez J, Pearson M, Sorenson BH, James B, Sambo C. Evaluation of the Health Results Innovation Trust Fund. 2012.

[23] World Bank. Harmonized List of Fragile Situations. Washington, DC: World Bank—http://www.worldbank.org/en/topic/fragilityconflictviolence/brief/harmonized-list-of-fragile-situations; 2016.

[24] OECD. States of Fragility 2015 [Internet]. Paris: Organisation for Economic Co-operation and Development; 2016. http://www.oecd-ilibrary.org/content/book/9789264227699-en

[25] Taylor S. Fragile and Conflict-Affected States: Exploring the Relationship Between Governance, Instability and Violence. Stab Int J Secur Dev. 2014;3.

[26] Pavignani E, Colombo S. Analysing Disrupted Health Sectors: a Modular Manual. Geneva: World Health Organization; 2009.

[27] Eldridge C, Palmer N. Performance-based payment: some reflections on the discourse, evidence and unanswered questions. Health Policy Plan. 2009;24: 160–6. doi: 10.1093/heapol/czp002 19202163 10.1093/heapol/czp002

[28] Pavignani E, Michael M, Murru M, Beesley ME, Hill PS. Making sense of the apparent chaos: health-care provision in six country case studies. Int Rev Red Cross. 2013;95: 41–60.

[29] Toonen J, Canavan A, Vergeer P, Elovainio R. Performance Based Financing: A Synthesis Report. Amsterdam: KIT, in collaboration with Cordaid and WHO; 2009.

[30] Woodward A, Sondorp E, Witter S, Martineau T. Health systems research in fragile and conflict-affected states: a research agenda-setting exercise. Heal Res Policy Syst. Health Research Policy and Systems; 2016;14: 51 doi: 10.1186/s12961-016-0124-1 27439611 10.1186/s12961-016-0124-1 PMC4955129

[31] Witter S. Health financing in fragile and post-conflict states: what do we know and what are the gaps? Soc Sci Med. 2012;75: 2370–2377. 23040083 10.1016/j.socscimed.2012.09.012

[32] Toonen J, Lodenstein E, Coolen A, Van der Wal B, Ambadire R, Guribie N, et al Results-Based Financing in healthcare Developing an RBF approach for healthcare in different contexts: the case of Mali and Ghana. Amsterdam: KIT; 2012.

[33] Soeters R, Griffiths F. Improving government health services through contract management: a case from Cambodia. Health Policy Plan. 2003;18: 10. 10.1093/heapol/18.1.74 12582110

[34] MSHP. Projet pilote de motivation basée sur la performance en Côte d’Ivoire. Abidjan: MSHP, USAID, EGPAF, Abt Assoc; 2010.

[35] Meessen B, Kashala JPI, Musango L. Output-based payment to boost staff productivity in public health centres: Contracting in Kabutare district, Rwanda. Bull World Health Organ. 2007;85: 108–115. doi: 10.2471/BLT.06.032110 17308731 10.2471/BLT.06.032110 PMC2636284

[36] Government of Burundi. Etats-Généraux de la Santé au Burundi. Bujumbura: Government of Burundi; 2005.

[37] Meessen B, Musango L, Kashala J-P. L’Initiative pour la Performance, Province de Butare, Rwanda. Butare: HealthNet International & Government of Rwanda; 2004.

[38] Republic of Congo. Manuel d’exécution de la strétgie de financement basé sur la performance. 2015.

[39] Remme M, Peerenboom PB, Douzima P-M, Batubenga DM, Inoussa MI, van de Weerd J. Le Financement basé sur la performance et la Bonne Gouvernance: Leçons apprises en République Centrafricaine. PBF CoP Working Paper Series—WP8; 2012.

[40] Basenya O, Nimpagaritse M, Busogoro F, Ndayishimiye J, Nkuzimana C, Ntahimpereye G, et al. Le financement basé sur la performance comme stratégie pour améliorer la mise en oeuvre de la gratuité des soins: premières leçons de l’expérience du Burundi. PBF CoP Working Paper Series; 2011.

[41] MSHP. Strategie Nationale de financement base sur la performance. Abidjan: Ministère de la Santé et de l’Hygiene Publique; 2014.

[42] MSSPSPG. Orientations stratégiques nationales du FBP aux Comores et cadrage institutionel. Ministère de la Santé, de la Solidarité, de la Protection Sociale et de la Promotion du Genre; 2016.

[43] Gautier L. Le financement basé sur les résultats au Mali—Note de Politique. 2016.

[44] Schramm N. Reflections from Sierra Leone: How Performance-Based (under) Financing Still Makes a Difference. World Bank RBF Health Blog; 2015.

[45] Murru M, Pavignani E. Democratic Republic of Congo: The chronically-ill heart of Africa Providing Health Care in Severely-Disrupted Environments A Multy-County Study. Brisbane: University of Queensland; 2012.

[46] Kiendrébéogo JA, Abdramane B, Lamoudi Y, Mahamat B, Shroff Z, Meessen B. Why Performance-Based Financing in Chad failed to emerge on the national policy agenda? Heal Syst Reforms. 2017;3: 80–90. doi: 10.1080/23288604.2017.1280115 10.1080/23288604.2017.1280115 31514677

[47] Manirambona J, Ntakarutimana L, Muhorane C, Fedjo G. La Transparence et la Redevabilité dans la Gestion de la Gratuité des Soins à Travers les Mécanismes de Financement Basé sur la Performance au Burundi. Bujumbura: Ministere de la Santé Publique; 2014.

[48] Chimhutu V, Tjomsland M, Songstad NG, Mrisho M, Moland KM. Introducing payment for performance in the health sector of Tanzania- the policy process. Global Health. Globalization and Health; 2015;11: 38 doi: 10.1186/s12992-015-0125-9 26330198 10.1186/s12992-015-0125-9 PMC4557903

[49] Shroff ZC, Bigdeli M, Meessen B. From Scheme to System (Part 2): Findings from Ten Countries on the Policy Evolution of Results-Based Financing in Health Systems. Heal Syst Reform. 2017;3: 137–147. doi: 10.1080/23288604.2017.1304190 10.1080/23288604.2017.1304190 31514674

[50] Keugoung B, Tsafack JP, Fouelifack FY, Sieleunou I, Ayissi Noubosse I, Boulenger D. Expérience pilote de financement basé sur la performance dans le Diocèse de Batouri au Cameroun: leçons pour l’extension du modèle. PBF CoP Working Paper Series—WP2; 2011.

[51] Meessen B, Kouanda S. Communities of practice: the missing link for knowledge management on implementation issues in low-income counies? Trop Med Int Heal. 2011;16: 1007–1014. doi: 10.1111/j.1365-3156.2011.02794.x 21564426 10.1111/j.1365-3156.2011.02794.x

[52] Peerenboom PB, Basenya O, Bossuyt M, Ndayishimiye J, Ntakarutimana L, van de Weerd J. La bonne gouvernance dans la réforme du financement du système de santé au Burundi. Sante Publique (Paris). 2014;2: 229–240. 25108965

[53] Sieleunou I, Taptue Fotso J-C, Kouokam E, Magne Tamga D, Azinyui Yumo H, Turcotte-Tremblay A-M, et al. Challenges of integrating an innovative health financing scheme into the health system: lessons from Performance-Based-Financing (PBF) in Cameroon (2006–2015). Antwerp & Geneva: Implementation Research: Taking Results Based Financing from scheme to system—research report; 2016.

[54] Sondorp E, Coolen A. The evolution of health service delivery in the Liberian health sector between 2003 and 2010. London & The Hague: LSHTM & KIT; 2012.

[55] Bold T, Collier P, Zeitlin A. The Provision of Social Services in Fragile States: Independent Service Authorities as a New Modality. Oxford: Centre for the Study of African Economies, University of Oxford; 2009.

[56] World Bank. Project Appraisal Document to the Republic of Cote d’Ivoire for a Health Systems Strengthening and Ebola Preparedness Project. World Bank, editor. Washington, DC; 2014.

[57] van de Looij F, Mureyi D, Sisimayi C, Koot J, Manangazira P, Musuka N. Early evidence from results-based financing in rural Zimbabwe. African Heal Monit. 2015;6: 32–36.

[58] Diongue B, Mayaka S, Mangala A. Rapport Final de la Mission d’Evaluation des Agences d’Achat des Performances des Services de Santé au Kassaï Occidental, au Sud et au Nord Kivu en République Démocratique du Congo. Kinshasa: KIT, Cordaid, HealthNetTPO, Programme Santé 9ème FED; 2008.

[59] Lafort Y, Letourny A, Koussémou A. Évaluation et capitalisation du Projet Santé 9ème FED. EU—Rapport final République Démocratique du Congo. Brussels: European Union; 2012.

[60] Bredenkamp C, De Borman N, Mullen P, Ostiguy D, Sompwe E, Wane W, et al. Dealing with difficult design decisions: The experience of an RBF pilot program in Haut- Katanga District of Democratic Republic of Congo. Washington, DC: World Bank—unpublished report; 2011.

[61] Rusa L, Fritsche G. Rwanda: performance-based financing in health Emerging Good Practice in Managing for Development Results: Sourcebook Manging for Development Results; 2013.

[62] Bertone MP, Samai M, Edem-Hotah J, Witter S. A window of opportunity for reform in post-conflict settings? The case of Human Resources for Health policies in Sierra Leone, 2002–2012. Confl Health. 2014;8: 11 doi: 10.1186/1752-1505-8-11 25075212 10.1186/1752-1505-8-11 PMC4114084

[63] Camara, Sidibé, Tamboula, Toonen J, Bulthuis. Le Rapport de Capitalisation de la phase pré-pilote du Financement Basé sur les Résultats (FBR) à Mamou, Guinée. Conakry: KIT; 2017.

[64] Banga-Mingo JP, Kossi-Mazouka A, Soeters R, Love J. Evaluation du Financement basé sur la Performance dans la Préfecture de Nana Mambéré pendant la crise humanitaire 2013–2014. The Hague: Cordaid; 2014.

[65] Shu Atanga J, Tsafack JP, Moussoume E, Kum Ghabowen I. How Performance Based Financing empowers the community and improves access to quality care in Eastern and North-Western Cameroon [Internet]. World Bank RBF Health; 2015. https://www.rbfhealth.org/sites/rbf/files/How%20PBF%20Empowers%20the%20Community%20in%20Cameroon.pdf

[66] PASS/MSHPP. Manuel d’execution du financement base sur la performance (FBP) en Republique Centrafricaine. Bangui: Projet d’Appui au Systeme de Sante—Ministere de la Santé, de l’Hygiene Publique et de la Population; 2017.

[67] Zombré D, De Allegri M, Ridde V. L’introduction puis le retrait du FBR n’ont pas eu d’effet sur l’utilisation des services de santé maternelle et infantile dans la région de Koulikoro au Mali—Note de Politique. 2017.

[68] Falisse J-B, Meessen B, Ndayishimiye J, Bossuyt M. Community Participation and Voice Mechanisms under Performance-Based Financing schemes in Burundi. Trop Med Int Heal. 2012;17: 674–682. doi: 10.1111/j.1365-3156.2012.02973.x 22487362 10.1111/j.1365-3156.2012.02973.x

[69] World Bank. Rewarding Provider Performance to Improve Quality and Coverage of Maternal and Child Health Outcomes Zimbabwe Results-Based Financing Pilot Program—Evidence to Inform Policy and Management Decisions. Washington, DC: World Bank; 2016.

[70] Zeng W, Cros M, Wright KD, Shepard DS. Impact of Performance-Based Financing on Primary Health Care Services in Haiti. Health Policy Plan. 2013;28: 596–605. doi: 10.1093/heapol/czs099 23107831 10.1093/heapol/czs099

[71] LSTM. Independent Evaluation of the Health Transition Fund in Zimbabwe. Liverpool: Centre for Maternal and Newborn Health—Liverpool School of Tropical Medicine; 2016.

[72] Toonen J. Rapport de la mission d’appui à Gao, sur l’approche de financement basé sur les resultats. 2017.

[73] Kiendrébéogo JA, Barthès O, Antony M, Rusa L. Piloting a performance-based financing scheme in Chad: Early results and lessons learned. African Heal Monit. 2015;7: 37–42.

[74] World Bank. Rapport de l’evaluation de l’experience pilote du financement base sur les resultats au Tchad. World Bank—unpublished report; 2013.

[75] Bertone MP, Lagarde M, Witter S. Performance-Based Financing in the context of the complex remuneration of health workers: findings from a mixed-method study in rural Sierra Leone. BMC Health Serv Res. 2016;16: 286 doi: 10.1186/s12913-016-1546-8 27435164 10.1186/s12913-016-1546-8 PMC4952280

[76] Chimhutu V, Songstad NG, Tjomsland M, Mrisho M, Moland KM. The inescapable question of fairness in Pay-for-performance bonus distribution: a qualitative study of health workers’ experiences in Tanzania. Global Health. Globalization and Health; 2016;12: 77 doi: 10.1186/s12992-016-0213-5 27884185 10.1186/s12992-016-0213-5 PMC5123229

[77] Ogundeji YK, Jackson C, Sheldon T, Olubajo O, Ihebuzor N. Pay for performance in Nigeria: the influence of context and implementation on results. Health Policy Plan. 2016;31: 955–963. doi: 10.1093/heapol/czw016 27036415 10.1093/heapol/czw016

[78] Bhatnagar A, George AS. Motivating health workers up to a limit: partial effects of performance-based financing on working environments in Nigeria. Health Policy Plan. 2016;31: 868–877. doi: 10.1093/heapol/czw002 26946273 10.1093/heapol/czw002

[79] Eichler R, Auxila P, Pollack J. Performance Based Reimbursement to Improve Impact: Evidence from Haiti. Boston, MA: USAID/MSH—LAC Health Sector Reform Initiative; 2000.

[80] Hyeladzira G, Mbunya S, Ihebuzor N, Olubajo L, Margwa P. Building Resilient Systems through Performance-Based Financing in Fragile & Conflict-affected States: Case of Insurgency Affected Districts in Adamawa State, Nigeria. Presentation at AfHEA 2016 conference; 2016.

[81] Olafsdottir AE, Mayumana I, Mashasi I, Njau I, Mamdani M, Patouillard E, et al Pay for performance: an analysis of the context of implementation in a pilot project in Tanzania. BMC Health Serv Res. 2014;14: 392 doi: 10.1186/1472-6963-14-392 25227620 10.1186/1472-6963-14-392 PMC4261877

[82] Fukuda-Parr S, Hulme D. International norm dynamics and the “end of poverty”: understanding the Millennium Development Goals. Glob Gov a Rev Multilater Int Organ. 2011;17: 17–36.

[83] Harmer A. Understanding change in global health policy: ideas, discourse and networks. Glob Public Health. 2011;6: 703–718. doi: 10.1080/17441692.2010.515236 20924870 10.1080/17441692.2010.515236

[84] Jensen C, McPake B, Jones A. Landscaping review part 3: Review of international health policy transfer literature Learning for Action Across Health Systems. Oxford: Oxford Policy Management; 2017.

[85] Brown T, Bell M. Imperial or postcolonial governance? Dissecting the genealogy of a global public health strategy. Soc Sci Med. 2008;67: 1571–1579. doi: 10.1016/j.socscimed.2008.07.027 18771835 10.1016/j.socscimed.2008.07.027

[86] Jones CM, Clavier C, Potvin L. Adapting public policy theory for public health research: A framework to understand the development of national policies on global health. Soc Sci Med. 2017;177: 69–77. doi: 10.1016/j.socscimed.2017.01.048 28161673 10.1016/j.socscimed.2017.01.048

[87] Kothari U. Authority and expertise: The professionalisation of international development and the ordering of dissent. Antipode. 2005;37: 425–446.

[88] Lee K, Goodman H. Global policy networks: the propagation of health care financing reform since the 1980s In: Lee K, Buse K, Fustukian S, editors. Health policy in a Globalising World. Cambridge: Cambridge University Press; 2002.

[89] Fitzgerald L, Ferlie E, Wood M, Hawkins C. Interlocking interactions, the diffusion of innovations in health care. Hum Relations. 2002;55: 1429–1449.

[90] Gautier L, Ridde V. Health financing policies in Sub-Saharan Africa: government ownership or donors’ influence? A scoping review of policymaking processes. Glob Heal Res Policy. Global Health Research and Policy; 2017;2: 23 doi: 10.1186/s41256-017-0043-x 29202091 10.1186/s41256-017-0043-x PMC5683243

[91] Nay O. International organisations and the production of hegemonic knowledge: How the World Bank and the OECD helped invent the fragile state concept. Third World Q. 2014;35: 210–231.

[92] Barnes A, Brown GW, Harman S, Papamichail A. African participation and partnership in financing: A case study in global health policy. EQUINET Discussion Paper 102; 2014.

[93] Scher D. The promise of Imihigo: Decentralized service delivery in Rwanda, 2006–2010 [Internet]. Princeton, NJ: Pricenton University—Innovations for Successful Societies; 2010 http://www.princeton.edu/successfulsocieties

[94] Kamuzinzi M. Imihigo: A hybrid model associating traditional and modern logics in public policy implementation in Rwanda. Int J African Renaiss Stud Inter-and Transdiscipl. 2016;11: 123–141.

[95] Purdeková A. “Even if I am not here, there are so many eyes”: surveillance and state reach in Rwanda. J Mod Afr Stud. 2011;49: 475–497.

[96] Rusa L, Schneidman M, Fritsche G, Musango L. Rwanda: Performance-based financing in the public sector In: Eichler R, Levine R, editors. Performance incentives for global health: potential and pitfalls. Washington, DC: Centre for Global Development; 2009.

[97] Paul E, Dramé ML, Kashala J, Ndema AE, Kounnou M, Aïssan JC, et al Performance-Based Financing to Strengthen the Health System in Benin: Challenging the Mainstream Approach. Int J Heal Policy Manag. 2017;6: 1–13. doi: 10.15171/ijhpm.2017.42 29325401 10.15171/ijhpm.2017.42 PMC5745866

