# Supplementary material for: Context matters (but how and why?) A hypothesis-led literature review of performance based financing in fragile and conflict-affected health systems
Source: PLoS One. 2018 Apr 3;13(4):e0195301. doi: 10.1371/journal.pone.0195301 (PMC5882151; doi:10.1371/journal.pone.0195301)
Supplement: S1 Table — (DOCX) [file pone.0195301.s001.docx]

**S1 Table: List of fragile and conflict-affected states included in the literature review**

|  | ***Low income countries under stress*** | | | ***Harmonized list of fragile situations*** | | | | | | | |
| --- | --- | --- | --- | --- | --- | --- | --- | --- | --- | --- | --- |
|  | **2007** | **2008** | **2009** | **2010** | **2011** | **2012** | **2013** | **2014** | **2015** | **2016** | **2017** |
| **Afghanistan** | Severe | core | core | core | yes | yes | Yes | yes | yes | yes | yes |
| **Angola** | Core | core | core | core | yes | yes | Yes |  |  |  |  |
| **Bosnia & Herzegovina** |  |  |  |  | yes | yes | Yes | yes | yes | yes |  |
| **Burundi** | Core | core | core | core | yes | yes | yes | yes | yes | yes | yes |
| **Cameroon** |  |  | marginal | marginal |  |  |  |  |  |  |  |
| **CAR** | Severe | core | Core | core | yes | yes | yes | yes | yes | yes | yes |
| **Cambodia** | Marginal | marginal | marginal |  |  |  |  |  |  |  |  |
| **Chad** | Core | core | Core | core | yes | yes | yes | yes | yes | yes | yes |
| **Comores** | Severe | core | Core | core | yes | yes | yes | yes | yes | yes | yes |
| **Congo Rep** | Core | core | Core | core | yes | yes | yes | yes |  |  |  |
| **Cote d'Ivoire** | Severe | core | Core | core | yes | yes | yes | yes | yes | yes | yes |
| **Djibouti** | Marginal | marginal | marginal | marginal |  |  |  |  |  |  | yes |
| **DR Congo** | Core | core | Core | core | yes | yes | yes | yes | yes | yes | yes |
| **Eritrea** | Core | core | Core | core | yes | yes | yes | yes | yes | yes | yes |
| **Gambia, The** | Marginal | marginal | marginal | marginal |  |  |  |  |  | yes | yes |
| **Georgia** |  |  |  | yes | yes | yes |  |  |  |  |  |
| **Guinea** | Core | core | Core | core | yes | yes | yes |  |  |  |  |
| **Guinea-Bissau** | Core | core | Core | core | yes | yes | yes | yes | yes | yes | yes |
| **Haiti** | Core | core | Core | core | yes | yes | yes | yes | yes | yes | yes |
| **Iraq** |  |  |  |  | yes | yes | yes | yes | yes | yes | yes |
| **Kiribati** |  | marginal | marginal | core | yes | yes | yes | yes | yes | yes | yes |
| **Kosovo** | Core | core | Core | marginal | yes | yes | yes | yes | yes | yes | yes |
| **Lao PDR** | Core | marginal | marginal |  |  |  |  |  |  |  |  |
| **Lebanon** |  |  |  |  |  |  |  |  |  | yes | yes |
| **Liberia** | Severe | core | Core | core | yes | yes | yes | yes | yes | yes | yes |
| **Lybia** |  |  |  |  |  |  | yes | yes | yes | yes | yes |
| **Madagascar** |  |  |  |  |  |  |  | yes | yes | yes | yes |
| **Malawi** |  |  |  |  |  |  |  | yes |  |  |  |
| **Mali** |  |  |  |  |  |  |  | yes | yes | yes | yes |
| **Mauritania** | Marginal |  |  |  |  |  |  |  |  |  |  |
| **Marshall Islands** |  |  |  |  |  | yes | yes | yes | yes | yes | yes |
| **Micronesia** |  |  |  |  |  | yes | yes | yes | yes | yes | yes |
| **Myanmar** | Severe | core | Core | core | yes | yes | yes | yes | yes | yes | yes |
| **Nigeria** | Marginal |  |  |  |  |  |  |  |  |  |  |
| **Nepal** |  |  |  |  | yes | yes | yes | yes |  |  |  |
| **Papua New Guinea** | Marginal | marginal |  |  |  |  |  |  |  |  | yes |
| **Sao Tome and Principe** | Marginal | core | Core | core | yes |  |  |  |  |  |  |
| **Sierra Leone** | Marginal | marginal | marginal | marginal | yes | yes | yes | yes | yes | yes | yes |
| **Solomon Islands** | Core | core | Core | core | yes | yes | yes | yes | yes | yes | yes |
| **Somalia** | Severe | core | Core | core | yes | yes | yes | yes | yes | yes | yes |
| **South Sudan** |  |  |  |  |  |  | yes | yes | yes | yes | yes |
| **Sudan** | Core | core | Core | core | yes | yes | yes | yes | yes | yes | yes |
| **Syria** |  |  |  |  |  |  | yes | yes | yes | yes | yes |
| **Tajikistan** |  |  | marginal | marginal | yes |  |  |  |  |  |  |
| **Timor Leste** | Core | core | core | core | yes | yes | yes | yes | yes | yes |  |
| **Togo** | Severe | core | core | core | yes | yes | yes | yes | yes | yes | yes |
| **Tonga** | Core | core | core | marginal |  |  |  |  |  |  |  |
| **Tuvalu** |  |  |  |  |  |  | yes | yes | yes | yes | yes |
| **Uzbekistan** | Core | core | marginal |  |  |  |  |  |  |  |  |
| **Vanuatu** | Marginal | marginal |  |  |  |  |  |  |  |  |  |
| **West Bank & Gaza** | Severe | core | core | core | yes | yes | yes | yes | yes | yes | yes |
| **Yemen** |  |  | marginal | marginal | yes | yes | yes | yes | yes | yes | yes |
| **Zimbabwe** | Severe | core | core | core | yes | yes | yes | yes | yes | yes | yes |

**Source**: World Bank (<http://www.worldbank.org/en/topic/fragilityconflictviolence/brief/harmonized-list-of-fragile-situations> )

Note: We excluded Malawi from the analysis, as it made it onto the FCAS list only briefly in 2014. On the other hand, Rwanda was included in our review and analysis, because it was one of the first countries to adopt PBF at a time when conflict was relatively recent, and because it has been presented in previous studies as a relevant case for examining PBF in a post-conflict setting (Toonen et al., 2012). Rwanda has been on the OECD’s list of fragile countries but not on the World Bank’s.
