# Supplementary material for: Context matters (but how and why?) A hypothesis-led literature review of performance based financing in fragile and conflict-affected health systems
Source: PLoS One. 2018 Apr 3;13(4):e0195301. doi: 10.1371/journal.pone.0195301 (PMC5882151; doi:10.1371/journal.pone.0195301)
Supplement: S2 Table — (DOCX) [file pone.0195301.s002.docx]

**S2 Table: PBF experiences in FCAS, by characteristics, funder and stage of development**

| **Country** | **Timing of PBF introduction** | **Stage** | **Funders** | **Implementers** | **Geographic coverage** | **Key sources *(full references at the bottom of the table)*** |
| --- | --- | --- | --- | --- | --- | --- |
| **Afghanistan** | 2003-2008 (BEHP)  2010-present (P4P) | Large scale contracting-out and contracting-in projects covering vast rural areas in the country. | World Bank (Health Results Innovation Trust Fund / HRITF), USAID, Cordaid | Multiple national and international NGOs (Cordaid, AHDS), and MoH’s Strengthening Mechanism Agency | Multiple entire provinces and rural areas – it was a MoH decision to leave provision of services there to contracted NGOs | [1–4] |
|  | 2012-2013 | Pilot | Dutch Government / EU | Cordaid, local NGO AHDS | Tirin Kowt, province Urozgan | https://www.cordaid.org/en/  projects/innovation-project- in-urozgan-provincie/ |
| **Burundi** | 2006-2010 | Pilot | Cordaid / EU | Cordaid | 2 provinces (Bubanza and Cankuzo) | [5,6] |
|  | 2006-2010 | Pilot | Dutch Government | HealthNet TPO | 1 province (Gitega) |  |
|  | 2008-2010 | Pilot | EU | Cordaid | 2 provinces (Bubanza and Cankuzo) |  |
|  | 2008-2010 | Pilot | Switzerland | Swiss TPH | 1 province (Ngozi) |  |
|  | 2008-2010 | Pilot | Belgium | Belgian Technical Cooperation | 1 province (Kirundo) |  |
|  | 2010-present | National level scale-up | Government of Burundi, EU, Belgium (BTC), World Bank (IDA), USAID | MoH, with support from partners (including Cordaid and BTC) | Entire country |  |
| **Cambodia** | 1997-2016 | 3 waves of PBF: (1). 1997--2002: an external contracting-in and contracting-out pilot (2). 2003--2009: ‘hybrid contracting’ (3). 2009 and ongoing: a uniform model of internal contracting | AdB, World Bank and Swiss Red cross In the first phase; World Bank, AdB, UNICEF and DFID, Belgian Technical Cooperation in phase 2; internal resources in phase 3 (approx. 40%), and other donors from previous phases | In the first phase, it was five INGOs - Health Net International, Enfants et Développement,  Save the Children Australia and the Association of Medical Doctors of Asia. Then designated MoH units to deliver services, against performance-based staff incentives | At the beginning only 5 operational district. Then PBF extended to the whole country only to rural areas (no contracting model has been applied to urban facilities) | [7–9] |
| **Cameroon** | 2004/2007-2012 | Small-scale pilot (extended in two waves) | Dutch bilateral cooperation | Cordaid | Two dioceses in the eastern region (Catholic facilities) | [10] |
|  | 2011/2012- present | Large pilot (plans for a national scale-up) | World Bank | International agencies (AEDES & Cordaid). Since 2015 purchasing role transferred to MoH + contracting, verification to  RFHPs (Regional Funds for Health Promotion) | 26 health districts, 4 in the Littoral, 4 in the North West, 4 in the South West, and all 14 in the East. | [11–13] |
| **Central African Republic (CAR)** | 2009-present | Pilot (two phases: 2009-2010 + 2010-2014) | Cordaid | Cordaid | Nana‐Mambéré Prefecture (region 2): up to 48 health units since 2010 | [14,15] |
|  |  | Pilot (extended in waves) | European Union (Fonds Bekou) | Cordaid | Various regions |  |
|  | 2016 (end of) – present | Pilot (to be scaled up nation-wide) | World Bank | International agencies (Cordaid + AEDES) | 14 districts across 5 regions | [16] |
| **Chad** | 2011- 2013 | Pilot | World Bank + (later: July-Dec 2013) Govt of Chad | International firm (AEDES/CSSI) | 8 districts in 4 regions | [17–20] |
| **Comoros** | 2011 – present | Pilot, extended gradually. Now with plans to scale-up to national level | Agence Française de Developpement (AfD) | International NGOs and firms (initially AEDES, 2011-2013) | Now covering Anjouan and Mohéli and 2 districts on Ngazidja | [21] |
| **Congo Rep** | 2012-2014 | Pilot | Cordaid + Memisa Belgium | Cordaid | 3 regions (Plateaux, Pool, and Niari): 73 health centres | [22] |
|  | 2016 – present | Large-scale implementation | US$ 100M, World Ministry of Health, IDA (US$ 10M), HRITF ( US$ 10M) | Ministry of Health | 7 departments: Niari, Plateaux, Bouenza, Cuvette, Brazzaville, Pointe-Noire, Pool | [23] |
| **Cote d’Ivoire** | 2006 - 2009 | Early pilot | PEPFAR | EGPAF (HIV focus) | (not specified) |  |
|  | 2009-2011 | Early pilot in conflict-affected North | PEPFAR | Abt Associates (HIV focus) | Ferkessédougou District | [24] |
|  | 2015-present | Pilot, to be extended in two waves | World Bank + govt of RCI | MoH/ international firm | Selected districts (initially 4) | [25] |
| **Djibouti** | 2014 – present | Pilot (to be extended in a second phase) | World Bank | MoH (external verification by international firm every quarter) | 2 regions (Sabieh and Tadjourah). | [26] |
| **DR Congo** | 2005-2010 | Pilot | PS9FED | Private – AEDES / FASS | Kasai Occidental and Oriental, Nord Kivu | [27] |
|  | 2005-2010 | Pilot | EU | HealthNet | Nord Kivu | [27] |
|  | 2006-2011 | Pilot | World Bank | PARSS | Bandundu, Maniema, Equateur, Katanga, Kinshasa | [28] |
|  | 2009-2013 | Pilot | World Bank / HRSP | NGO | Haut Katanga District | [29] |
|  | 2006- | Pilot / some districts only | Cordaid | Cordaid | Sud Kivu | [30] |
|  | 2008- | Pilot / some districts only | Cordaid | Cordaid | Bas Congo | https://www.cordaid.org/ |
|  | 2017- | Scale up | Global Fund, World Bank, UNICEF | Ministry of Health through EUP | Katanga, Equateur, Maniema, Bandundu |  |
| **The Gambia** | 2014-2015 | Pilot | World Bank | National Nutrition Agency (NaNA) and the Ministry of Health and Social Welfare (MOHSW) | North Bank West region | [31] |
|  | 2015 – present | Extension | World Bank | National Nutrition Agency (NaNA) and the Ministry of Health and Social Welfare (MOHSW) | Central River, North Bank West and Upper River regions. | [31] |
| **Guinea** | 2016 – present | Pre-pilot (FBR+) to be extended in 2017 with multi-donor funding | World Bank (funding indicators)  +Dutch (funding TA/implementation) | Health Focus (KIT+HDP) | Mamou District | [32] |
| **Guinea Bissau** | 2013-2016  and ongoing  (Programa Integral de saúde Materno Infantil – Integrated Mother and Child Health Programme) | Implemented large scale (entire provinces) and in evaluation phase | European Commission, UNICEF | Portuguese NGOs (Instituto Marquês Vale Flor, Vida, EMI) | 5 regions out of 11, covering 40% of overall country population (all rural) | [33] |
| **Haiti** | 1996-1998 Pilot project funded by IADB  1999-2001  2005-2009  ongoing (end in 2018) | First piloted in 3 health facilities. Then scaled up to 27 facilities and respective NGOs. Today PBF covers 3 Departments and 1.8 million people. | USAID with the support of MSH in the first phase. Now supported also by World Bank (HRITF). | US NGOs | Three Departments | [34] |
|  | 2014-present | Pilot – scale up process started in April 2016 (some setbacks reported following Hurricane Matthew in 2016 that destroyed the information system) | World Bank, Global Fund and USAID | MoH + international agencies (MSH, JHIPEGO) | Initially launched in the North-eastern Department, then gradually scaled up. Now covers 7 Departments (only 3 Departments are not included) | [35] |
| **Lao PDR** | 2001-2008 | District health management systems – Provincial Health for Nambak District | Swiss Red Cross; Belgian Technical Cooperation; Lao-Luxembourg | Contracting-in with RBF from project to district health and facilities and performance based incentives for the staff | Initially launched in Nambak district from 2001-2005, it was subsequently expanded to 3 more provinces | [36] |
|  | 2014-2016 | RBF Pay for Quality | Initially MoH and World Bank, then Swiss Red Cross | Result Based Financing schemes Pay-for-Quality (PFQ) and Pay-For-Preventive schemes (PFP) | 3 provinces | [37] |
| **Liberia** | 2011-present | Mixed approach: contracting-in (MoH/CHTs) + management contracting (MoH/NGOs) + PBF (NGOs/HFs) | USAID (+ others supporting MoH’s PBF Unit) | Contracted implementers (CHTs and/or NGOs) | 3 counties (Bong, Lofa, and Nimba) | [38,39] |
|  | 2013-present | Pilot at hospital/  secondary level (6 facilities) | World Bank | MoH (international firm for verification) | Redemption Hospital. Scaled-up to five extra hospitals in 2016 (Phebe, CB Dunbar, Tellewoyan, Jackson F. Doe, F.J. Grant) | [40] |
| **Mali** | 2012- 2013 | Pre-pilot (“OMD5/FBR”) | MoH / MDG5 Trust Fund (funded by Dutch Embassy) | NGOs (KIT, SNV) | 3 districts in Koulikoro region | [41,42] |
|  | 2016-2017 | Pilot (“PRSR/FBR”) | World Bank | Consortium KIT-CGIC-Cordaid (transition to national body envisaged for the future) | All 10 districts in Koulikoro region | [41–43] |
|  | 2017-present | Design stage |  | MdM Belgium | Gao region, Districts of Gao and Bourem (conflict-affected North) |  |
| **Nigeria** | 2011-present | Pilot (pre-pilot from Dec 2011 to Dec 2012) | World Bank | State Primary Health Care Development Agency/Board (SPHCDA/B) + international firm contracted as purchaser | Ondo, Nasarawa and Adamawa States (Adamawa affected by insurgency 2011-2015) | [44] |
| **Rwanda** | 2002-2008 | Pilot | SIDA | HealthNet TPO | Butare | [45,46] |
|  | 2002-2008 | Pilot | Memisa/Cordaid | Cordaid | Cyangugu | [46,47] |
|  | 2005-2008 | Pilot | Belgium | BTC | Kigali-Ngali, Kabgayi, Kigali Ville | [46,47] |
|  | 2008- | National level | Government, with the World Bank, USAID, and the Global Fund (also BTC, HNI and Cordaid earlier) | Ministry of education | Nationwide | [46,47] |
| **Sierra Leone** | 2011-2015 | National-level project | World Bank | MoHS | Country-wide in primary facilities + pilot in two tertiary hospitals in Freetown | [48,49] |
|  | 2015 (March to October) | Pilot (“PBF Plus”) | Cordaid/World Bank | MoHS /Cordaid | One district (Bombali) | [50] |
| **Tajikistan** | 2014 - present | Pilot in eight rayons (districts) | World Bank (HIRTF) | MoH | Eight rayons (districts) in Khatlon and Sughd oblasts (regions) | [51,52] |
| **Zimbabwe** | 2011 – present | Pilot, extended in several waves | World Bank | Cordaid (as NPA, National Purchasing Agency) | 2 districts (Zvishavane+  Marondera). Later extended to 18 rural districts | [53–55] |
|  | 2014-present | National scale-up (rural areas) | Health Transition Fund/Health Development Fund (multi-donor, administered by UNICEF) | MoHCC + Crown Agents as NPA | Remaining 42 districts | [55,56] |

**References for Table S2**

1. Alonge O, Gupta S, Engineer C, Salehi AS, Peters DH. Assessing the pro-Poor Effect of Different Contracting Schemes for Health Services on Health Facilities in Rural Afghanistan. Health Policy Plan. 2015;30: 1229–42.

2. Arur A, Peters D, Hansen P, Mashkoor MA, Steinhardt LC, Burnham G. Contracting for Health and Curative Care Use in Afghanistan between 2004 and 2005. Health Policy Plan. 2010;25: 135–144.

3. Engineer CY, Dale E, Agarwal A, Agarwal A, Alonge O, Edward A, et al. Effectiveness of a Pay-for-Performance Intervention to Improve Maternal and Child Health Services in Afghanistan: A Cluster-Randomized Trial. Int J Epidemiol. 2016;45: 451–59.

4. Salama P, Alwan A. Building Health Systems in Fragile States: The Instructive Example of Afghanistan. Lancet Glob Heal. 2016;4: e351–52.

5. Bonfrer I, Van de Poel E, Van Doorslaer E. The effects of performance incentives on the utilization and quality of maternal and child care in Burundi. Soc Sci Med. 2014;123: 96–104.

6. Falisse J-B, Ndayishimiye J, Kamenyero V, Bossuyt M. Performance-based financing in the context of selective free health-care: an evaluation of its effects on the use of primary health-care services in Burundi using routine data. Health Policy Plan. 2014;30: 1251–1260. doi:10.1093/heapol/czu132

7. Jacobs B, Thomé J-M, Overtoom R, Sam SO, Indermühle L, Price N. From Public to Private and Back Again: Sustaining a High Service-Delivery Level during Transition of Management Authority: A Cambodia Case Study. Health Policy Plan. 2010;25: 197–208.

8. Khim K, Annear PL. Strengthening District Health Service Management and Delivery through Internal Contracting: Lessons from Pilot Projects in Cambodia. Soc Sci Med. 2013;96: 241–49.

9. Khim K, Ir P, Annear PL. Factors Driving Changes in the Design, Implementation, and Scaling-Up of the Contracting of Health Services in Rural Cambodia, 1997–2015. Heal Syst Reform. 2017;3: 105–16.

10. Keugoung B, Tsafack JP, Fouelifack FY, Sieleunou I, Ayissi Noubosse I, Boulenger D. Expérience pilote de financement basé sur la performance dans le Diocèse de Batouri au Cameroun: leçons pour l’extension du modèle. PBF CoP Working Paper Series - WP2; 2011.

11. AEDES-IRESCO. Performance Based Financing Implementation Procedures. Manual for the North-West Region of Cameroon. 2012.

12. Sieleunou I, Taptue Fotso J-C, Kouokam E, Magne Tamga D, Azinyui Yumo H, Turcotte-Tremblay A-M, et al. Challenges of integrating an innovative health financing scheme into the health system: lessons from Performance-Based-Financing (PBF) in Cameroon (2006 - 2015). Antwerp & Geneva: Implementation Research: Taking Results Based Financing from scheme to system - research report; 2016.

13. Sieleunou I, Turcotte-Tremblay A-M, Yumo HA, Kouokam E, Taptue Fotso J-C, Magne Tamga D, et al. Transferring the Purchasing Role from International to National Organizations During the Scale-Up Phase of Performance-Based Financing in Cameroon. Heal Syst Reform. 2017;3: 91–104. doi:10.1080/23288604.2017.1291218

14. Banga-Mingo JP, Kossi-Mazouka A, Soeters R, Love J. Evaluation du Financement basé sur la Performance dans la Préfecture de Nana Mambéré pendant la crise humanitaire 2013-2014. The Hague: Cordaid; 2014.

15. Remme M, Peerenboom PB, Douzima P-M, Batubenga DM, Inoussa MI, van de Weerd J. Le Financement basé sur la performance et la Bonne Gouvernance : Leçons apprises en République Centrafricaine. PBF CoP Working Paper Series - WP8; 2012.

16. PASS/MSHPP. Manuel d’execution du financement base sur la performance (FBP) en Republique Centrafricaine. Bangui: Projet d’Appui au Systeme de Sante - Ministere de la Santé, de l’Hygiene Publique et de la Population; 2017.

17. Kiendrébéogo JA, Abdramane B, Lamoudi Y, Mahamat B, Shroff Z, Meessen B. Why Performance-Based Financing in Chad failed to emerge on the national policy agenda? Heal Syst Reforms. 2017;3: 80–90. doi:10.1080/23288604.2017.1280115

18. Kiendrébéogo JA, Barthès O, Antony M, Rusa L. Piloting a performance-based financing scheme in Chad: Early results and lessons learned. African Heal Monit. 2015;7: 37–42.

19. World Bank. Rapport de l’evaluation de l’experience pilote du financement base sur les resultats au Tchad. World Bank - unpublished report; 2013.

20. MPECI/MSP. Manuel des procedures pour la mise en œuvre du financement basé sur les résultats au Tchad [Internet]. N’djamena: Ministere du Plan, de l’Economie et de la Cooperation Internationale, Ministere de la Santé Publique; 2011. Available: http://santeplusburundi.org/images/PDF/manuelfbp042010.pdf

21. MSSPSPG. Manuel de procédures du Financement Basé sur la Performance aux Comores. Ministère de la Santé, de la Solidarité, de la Protection Sociale et de la Promotion du Genre; 2016.

22. Cordaid. PBF in the Republic of Congo. Powerpoint presentation; 2013.

23. Republic of Congo. Manuel d’exécution de la strétgie de financement basé sur la performance. 2015.

24. MSHP. Projet pilote de motivation basée sur la performance en Côte d’Ivoire. Abidjan: MSHP, USAID, EGPAF, Abt Assoc; 2010.

25. MSHP. Strategie Nationale de financement base sur la performance. Abidjan: Ministère de la Santé et de l’Hygiene Publique; 2014.

26. MS. Manuel des procédures pour la mise en œuvre du financement base sur la performance à Djibouti. Djibouti: Ministère de la Santé; 2014.

27. Lafort Y, Letourny A, Koussémou A. Évaluation et capitalisation du Projet Santé 9ème FED. EU - Rapport final République Démocratique du Congo. Brussels: European Union; 2012.

28. Bredenkamp C, De Borman N, Mullen P, Ostiguy D, Sompwe E, Wane W, et al. Dealing with difficult design decisions: The experience of an RBF pilot program in Haut- Katanga District of Democratic Republic of Congo. Washington, DC: World Bank - unpublished report; 2011.

29. Murru M, Pavignani E. Democratic Republic of Congo: The chronically-ill heart of Africa. Providing Health Care in Severely-Disrupted Environments A Multy-County Study. Brisbane: University of Queensland; 2012.

30. Diongue B. Rapport Final de la Mission d’Evaluation des Agences d’Achat des Performances des Services de Santé au Kassaï Occidental, au Sud et au Nord Kivu en République Démocratique du Congo. Amsterdam: KIT; 2008.

31. MHSW. Maternal and Child Nutrition and Health Results Project (MCNHRP) - Project Operations Manual. Banjul: Ministry of Health and Social Welfare; 2015.

32. Camara, Sidibé, Tamboula, Toonen J, Bulthuis. Le Rapport de Capitalisation de la phase pré-pilote du Financement Basé sur les Résultats (FBR) à Mamou, Guinée. Conakry: KIT; 2017.

33. Manzanares A, Eguiluz R. Programa Integral de Saúde Materno Infantil. Relatório Final de Avaliação. Bissau: Europe Aid; 2016.

34. Eichler R, Auxila P, Pollack J. Performance Based Reimbursement to Improve Impact: Evidence from Haiti. Boston, MA: USAID/MSH - LAC Health Sector Reform Initiative; 2000.

35. Zeng W, Cros M, Wright KD, Shepard DS. Impact of Performance-Based Financing on Primary Health Care Services in Haiti. Health Policy Plan. 2013;28: 596–605.

36. MoH. Health Services Improvement Project. Operational guidelines. Vientiane: Ministry of Health, Lao-PDR; 2014.

37. World Bank. Implementation completion and results report to the Lao PDR for a health services improvement project. Washington, DC: World Bank - Health, Nutrition, and Population Global Practice East Asia and Pacific Region; 2016.

38. Petit D, Sondorp E, Mayhew S, Roura M, Roberts B. Implementing a Basic Package of Health Services in post-conflict Liberia: Perceptions of key stakeholders. Soc Sci Med. Elsevier Ltd; 2013;78: 42–9. doi:10.1016/j.socscimed.2012.11.026

39. Sondorp E, Coolen A. The evolution of health service delivery in the Liberian health sector between 2003 and 2010. London & The Hague: LSHTM & KIT; 2012.

40. MoH. Performance-Based Financing Operational Manual. Monrovia: Ministry of Health; 2016.

41. Gautier L. Le financement basé sur les résultats au Mali - Note de Politique. 2016.

42. Zombré D, De Allegri M, Ridde V. L’introduction puis le retrait du FBR n’ont pas eu d’effet sur l’utilisation des services de santé maternelle et infantile dans la région de Koulikoro au Mali - Note de Politique. 2017.

43. MS/WB. Manuel Opérationnel du Financement basé sur les résultats du projet PRSR-FBR. Ministère de la Santé, Banque Mondiale, KIT-CGIC-Cordaid; 2016.

44. Hyeladzira G, Mbunya S, Ihebuzor N, Olubajo L, Margwa P. Building Resilient Systems through Performance-Based Financing in Fragile & Conflict-affected States: Case of Insurgency Affected Districts in Adamawa State, Nigeria. Presentation at AfHEA 2016 conference; 2016.

45. Meessen B, Musango L, Kashala J-P. L’Initiative pour la Performance, Province de Butare, Rwanda. Butare: HealthNet International & Government of Rwanda; 2004.

46. Rusa L, Fritsche G. Rwanda: performance-based financing in health. Emerging Good Practice in Managing for Development Results: Sourcebook. Manging for Development Results; 2013.

47. Basinga P, Gertler PJ, Binagwaho A, Soucat AL, Sturdy J, Vermeersch CM. Effect on maternal and child health services in Rwanda of payment to primary health-care providers for performance: an impact evaluation. Lancet. 2011;377: 1421–1428.

48. Schramm N. Reflections from Sierra Leone: How Performance-Based (under) Financing Still Makes a Difference. World Bank RBF Health Blog; 2015.

49. MoHS. Sierra Leone Simple Performance Based Financing Scheme for Primary Healthcare - Operational Manual 2011. Freetown: Ministry of Health and Sanitation; 2011.

50. Hakuzimana A, Kruijzen M, van de Looij F, van ‘T Riet H, Oranje M, Schramm N. Findings from the PBF Plus Pilot. Freetown: Cordaid; 2015.

51. MoH. Project Implementation Manual. Tajikistan Performance-Based Financing Pilot Health Sector Improvement Project (HSIP). Dushambe: MoH - Tajikistan; 2013.

52. World Bank. Republic of Tajikistan. Feasibility Study for Results-Based Financing (RBF) in the Health Sector. Washington, DC: World Bank - Human Development Sector Unit Central Asia Country Unit Europe and Central Asia ( Report No. 53743-TJ); 2010.

53. van de Looij F, Mureyi D, Sisimayi C, Koot J, Manangazira P, Musuka N. Early evidence from results-based financing in rural Zimbabwe. African Heal Monit. 2015;6: 32–36.

54. World Bank. Rewarding Provider Performance to Improve Quality and Coverage of Maternal and Child Health Outcomes. Zimbabwe Results-Based Financing Pilot Program - Evidence to Inform Policy and Management Decisions. Washington, DC: World Bank; 2016.

55. MoHCC. National Results Based Financing Approach: Programme Implementation Manual. Harare: The Ministry of Health and Child Care; 2016.

56. LSTM. Independent Evaluation of the Health Transition Fund in Zimbabwe. Liverpool: Centre for Maternal and Newborn Health - Liverpool School of Tropical Medicine; 2016.
